# Correction: Virus Infection of Plants Alters Pollinator Preference: A Payback for Susceptible Hosts?

**DOI:** 10.1371/journal.ppat.1005906

**Published:** 2016-09-15

**Authors:** The PLOS Pathogens Staff

## Notice of Republication

This article was republished on August 30, 2016 to correct errors that were introduced during the typesetting process. The publisher apologizes for the errors. Please download this article again to view the correct version. The originally published, uncorrected article and the republished, corrected articles are provided here for reference.

## Supporting Information

S1 File Originally published, uncorrected article. (PDF)

S2 File Republished, corrected article. (PDF)
