# Supplementary material for: Virus Infection of Plants Alters Pollinator Preference: A Payback for Susceptible Hosts?
Source: PLoS Pathog. 2016 Aug 11;12(8):e1005790. doi: 10.1371/journal.ppat.1005790 (PMC4981420; doi:10.1371/journal.ppat.1005790)
Supplement: S1 Text — (PDF) [file ppat.1005790.s003.pdf]

Text S1. Full derivation of the mathematical model

1 Introduction

This document gives a more detailed derivation of the mathematical model.

2 Assumptions

The following assumptions were made in constructing the model.

1. The host plant population remains of constant size in all generations.
2. The population is large enough that a deterministic genetic model is appropriate.
3. We model a broad host range virus, which allows us to assume a constant proportion of virus susceptible plants become infected in each generation.
4. Infection occurs before flowers become reproductively mature.
5. Infection affects all infected plants equally.
6. Infection leads to reduced seed set by infected female (i.e. seed producing) parents.
7. There are no other deleterious effects of virus infection.
8. Pollinator density is constant in all generations, and this leads to a fixed number of total visits by pollinators to the plant population in each generation.
9. Flowers on infected plants are more likely to be visited by pollinators.
10. A visit by a pollinator always leads to pollination (either outcrossing due to pollen carried from other plants, or selfing due to the buzz effect and/or geitonogamy).
11. Pollinators carry pollen in amounts proportional to the fraction of visits to plants of each genotype.
12. A fraction of the ovules in flowers that are not visited by pollinators will self-pollinate.
13. There is no in-breeding depression (i.e. no fitness cost of self-pollination).
14. There is no cost of virus resistance.
15. Virus resistance is controlled by a single bi-allelic locus.
16. Virus resistance is genetically dominant over virus sensitivity.

3 Parameters and state variables

3.1 Input parameters

The model requires the following six input parameters.

- ν = pollinator bias to preferentially visit flowers on infected plants.
- α = probability that a virus susceptible plant becomes infected.
- δ = proportional number of viable seeds set by an infected female parent.
- γ = mean number of pollinator visits per flower (set by pollinator density).
- σ = probability that an ovule on a flower that is not visited by a pollinator self-pollinates.
- ϕ = probability that an ovule on a flower that is visited by a pollinator cross-pollinates.

Note that δ is a composite parameter that accounts for any differences between infected and uninfected plants in terms of: i) probability of pollinated flowers bearing fruit; ii) number of seeds per fruit; iii) probability of seed germination; and iv) probability of progeny surviving until reproductive maturity.

3.2 State variables tracked in the model

The model tracks the following state variables in each generation (n); each state variable is the relevant proportion of the host population that is of each genotype in generation n.

- x_n = homozygous virus resistant (genotype = RR; phenotype = R).
- y_n = heterozygous virus resistant (genotype = Rr; phenotype = R).
- z_n = homozygous virus susceptible (genotype = rr; phenotype = r).

4 Modelling the consequences of pollinator behaviour

4.1 Pollinator preference

A proportion α of virus susceptible plants become infected, and a proportion z_n of plants are virus susceptible, which means that

$$p_- = \mathbb{P}(\text{randomly chosen plant is uninfected}) = x_n + y_n + (1-\alpha)z_n = 1 - \alpha z_n, \quad (1)$$
$$p_+ = \mathbb{P}(\text{randomly chosen plant is infected}) = \alpha z_n. \quad (2)$$

Since ν is the bias shown by pollinators towards infected plants, and because each infected and uninfected plant is assumed to have the same number of flowers, individual pollinator visits

follow (see for e.g. [1], in which the same form is used in an epidemiological model of the bias shown by mosquitoes to bite humans who are infected with malaria)

$$\rho_- = \mathbb{P}(\text{pollinator chooses a flower on an uninfected plant on single visit}) = \frac{p_-}{p_- + \nu p_+}, \quad (3)$$

$$\rho_+ = \mathbb{P}(\text{pollinator chooses a flower on an infected plant on single visit}) = \frac{\nu p_+}{p_- + \nu p_+}. \quad (4)$$

Noting that $p_- + \nu p_+ = 1 + (\nu - 1)\alpha z_n$, and introducing the amalgamated parameter

$$\eta = \frac{1}{1 + (\nu - 1)\alpha z_n}, \quad (5)$$

the probabilities can be written more compactly as

$$\rho_- = \frac{1 - \alpha z_n}{1 + (\nu - 1)\alpha z_n} = (1 - \alpha z_n)\eta, \quad (6)$$

$$\rho_+ = \frac{\nu \alpha z_n}{1 + (\nu - 1)\alpha z_n} = \nu \alpha z_n \eta. \quad (7)$$

4.2 Probability that the male parent comes from each genotype

Define

$$\beta_g = \mathbb{P}(\text{male parent in an outcrossing is of genotype } g \in \{RR, Rr, rr\}). \quad (8)$$

Since pollinators are assumed to carry pollen in proportion to the visits they make to flowers, the value of β_g is controlled by the probability that a single pollinator visit is to a flower on a plant of each genotype. These probabilities can be calculated by conditioning the genotype on the infection status of the plants the pollinators visit (where + is infected, and − is uninfected)

$$\beta_{RR} = \mathbb{P}(RR|+)\rho_+ + \mathbb{P}(RR|-)\rho_-, \quad (9)$$

$$= 0 \times \nu \alpha z_n \eta + \frac{x_n}{1 - \alpha z_n} \times (1 - \alpha z_n)\eta, \quad (10)$$

$$= \eta x_n, \quad (11)$$

$$\beta_{Rr} = \mathbb{P}(Rr|+)\rho_+ + \mathbb{P}(Rr|-)\rho_-, \quad (12)$$

$$= 0 \times \nu \alpha z_n \eta + \frac{y_n}{1 - \alpha z_n} \times (1 - \alpha z_n)\eta, \quad (13)$$

$$= \eta y_n, \quad (14)$$

$$\beta_{rr} = \mathbb{P}(rr|+)\rho_+ + \mathbb{P}(rr|-)\rho_-, \quad (15)$$

$$= 1 \times \nu \alpha z_n \eta + \frac{(1 - \alpha) z_n}{1 - \alpha z_n} \times (1 - \alpha z_n)\eta, \quad (16)$$

$$= \eta(1 + (\nu - 1)\alpha) z_n. \quad (17)$$

As required, $\beta_{RR} + \beta_{Rr} + \beta_{rr} = 1$.

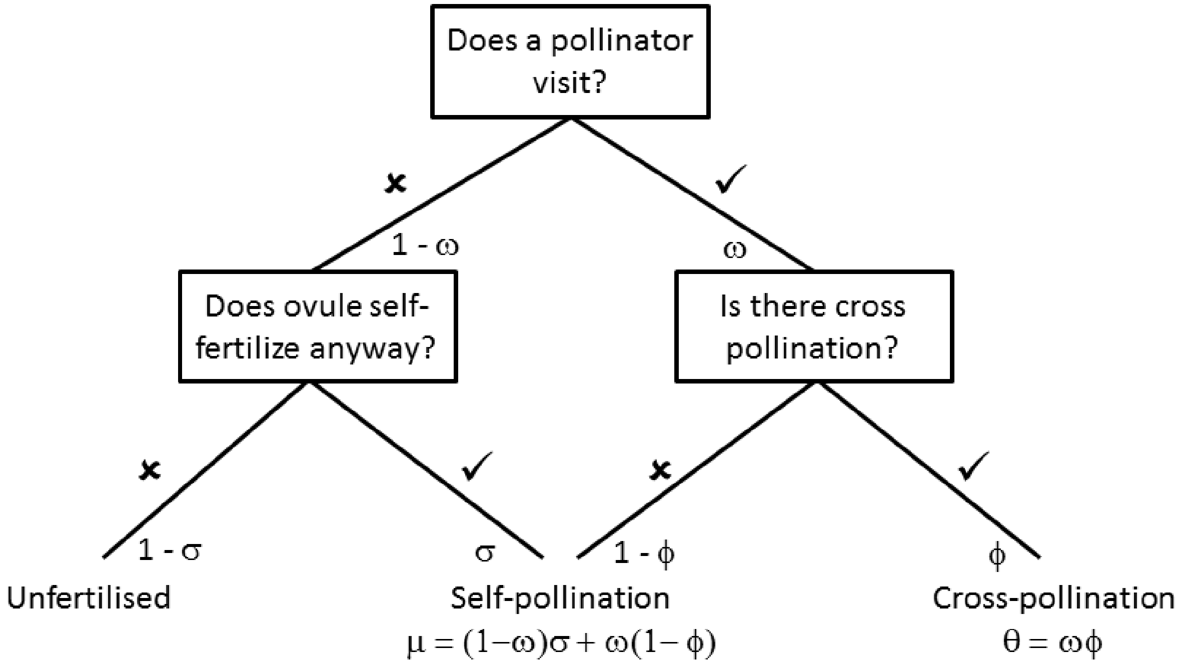

Figure 1: Schematic of the model for the fate of a single ovule. Note that ω is the probability that the flower is visited by a pollinator, and so depends on infection status.

4.3 Female side of the model

Consider (over the entire period that pollination is possible)

$$\gamma_- \;=\; \text{mean number of pollinator visits per flower on an uninfected plant,} \tag{18}$$

$$\gamma_+ \;=\; \text{mean number of pollinator visits per flower on an infected plant,} \tag{19}$$

where the pollinator bias means that

$$\gamma_+ \;=\; \nu\gamma_-. \tag{20}$$

Since the mean number of visits per flower over the entire plant population can be partitioned by infection status,

$$\gamma \;=\; \gamma_- p_- + \gamma_+ p_+, \tag{21}$$

it follows that

$$\gamma_- \;=\; \frac{\gamma}{1+(\nu-1)\alpha z_n} = \gamma\eta, \tag{22}$$

$$\gamma_+ \;=\; \frac{\nu\gamma}{1+(\nu-1)\alpha z_n} = \nu\gamma\eta. \tag{23}$$

Assuming that the number of visits received by an individual flower follows a Poisson distribution, the probability that an individual flower is visited at least once therefore depends on the infection status of the plant on which it is located

$$\omega_- \;=\; \mathbb{P}(\text{individual flower on uninfected plant is visited}) = 1 - e^{-\gamma\eta}, \tag{24}$$

$$\omega_+ \;=\; \mathbb{P}(\text{individual flower on infected plant is visited}) = 1 - e^{-\nu\gamma\eta}. \tag{25}$$

Note that these expressions account for the fact that, if there are fewer infected plants, flowers on uninfected plants will get proportionately more visits.

To model the effects of pollinator behaviour on reproduction, we assume [2, 3] (Figure 1)

$$\begin{aligned} \theta_- &= \mathbb{P}(\text{ovule on uninfected plant self-pollinates}) = (1-\omega_-)\sigma + \omega_-(1-\phi), && (26) \\ \mu_- &= \mathbb{P}(\text{ovule on uninfected plant cross-pollinates}) = \omega_-\phi, && (27) \end{aligned}$$

and

$$\begin{aligned} \theta_+ &= \mathbb{P}(\text{ovule on infected plant self-pollinates}) = (1-\omega_+)\sigma + \omega_+(1-\phi), && (28) \\ \mu_+ &= \mathbb{P}(\text{ovule on infected plant cross-pollinates}) = \omega_+\phi, && (29) \end{aligned}$$

The genetic model requires the proportionate reproduction of females on a phenotype by phenotype basis. Recalling that a proportion α of virus susceptible plants are infected, and that these females set a proportion δ of viable seed in comparison with uninfected plants, the relevant quantities are given by

$$\begin{aligned} \epsilon_R &= \text{relative reproduction of phenotype R via self-pollination} && (30) \\ &= \theta_-, && (31) \\ \kappa_R &= \text{relative reproduction of phenotype R via cross-pollination} && (32) \\ &= \mu_-, && (33) \\ \epsilon_r &= \text{relative reproduction of phenotype r via self-pollination} && (34) \\ &= \alpha\delta\theta_+ + (1-\alpha)\theta_-, && (35) \\ \kappa_r &= \text{relative reproduction of phenotype r via cross-pollination} && (36) \\ &= \alpha\delta\mu_+ + (1-\alpha)\mu_-. && (37) \end{aligned}$$

5 Evolutionary dynamics

5.1 Female parents of genotype RR

If the female (seed) parent is of genotype RR, then progeny from selfing are always of genotype RR. For outcrossing, the genotype of the progeny depends on that of the male (pollen) parent (Table 1). If $x_{n+1}(x_n)$ is defined to be the proportion of plants that are in class x in generation $n+1$ that have female parents in class x in generation n, then, since the female parents have the R phenotype

$$\begin{aligned} x_{n+1}(x_n) &= \zeta_n x_n \left(\epsilon_R + \kappa_R \left(\beta_{RR} + \frac{1}{2}\beta_{Rr}\right)\right), && (38) \\ y_{n+1}(x_n) &= \zeta_n x_n \left(\kappa_R \left(\frac{1}{2}\beta_{Rr} + \beta_{rr}\right)\right), && (39) \\ z_{n+1}(x_n) &= 0, && (40) \end{aligned}$$

where ζ_n is a scale factor set by the total amount of reproduction that will be fixed later to ensure the population size is constant over generations.

		Male		
		x	y	z
Female	x	all x	$\frac{1}{2}x{:}\frac{1}{2}y$	all y
	y	$\frac{1}{2}x{:}\frac{1}{2}y$	$\frac{1}{4}x{:}\frac{1}{2}y{:}\frac{1}{4}z$	$\frac{1}{2}y{:}\frac{1}{2}z$
	z	all y	$\frac{1}{2}y{:}\frac{1}{2}z$	all z

Table 1: Mendelian dependence of the genotype of offspring plants on the genotypes of female (seed producing) and male (pollen donor) parents.

5.2 Female parents of genotype Rr

The contributions of these female parents to the next generation are

$$x_{n+1}(y_n) = \zeta_n y_n \left(\frac{1}{4}\epsilon_R + \kappa_R \left(\frac{1}{2}\beta_{RR} + \frac{1}{4}\beta_{Rr} \right) \right), \tag{41}$$

$$y_{n+1}(y_n) = \zeta_n y_n \left(\frac{1}{2}\epsilon_R + \kappa_R \left(\frac{1}{2}\beta_{RR} + \frac{1}{2}\beta_{Rr} + \frac{1}{2}\beta_{rr} \right) \right) = \zeta_n y_n \left(\frac{1}{2}\left(\epsilon_R + \kappa_R\right) \right), \tag{42}$$

$$z_{n+1}(y_n) = \zeta_n y_n \left(\frac{1}{4}\epsilon_R + \kappa_R \left(\frac{1}{4}\beta_{Rr} + \frac{1}{2}\beta_{rr} \right) \right). \tag{43}$$

5.3 Female parents of genotype rr

The contributions of these female parents to the next generation are

$$x_{n+1}(z_n) = 0, \tag{44}$$

$$y_{n+1}(z_n) = \zeta_n z_n \left(\kappa_r \left(\beta_{RR} + \frac{1}{2}\beta_{Rr} \right) \right), \tag{45}$$

$$z_{n+1}(z_n) = \zeta_n z_n \left(\epsilon_r + \kappa_r \left(\frac{1}{2}\beta_{Rr} + \beta_{rr} \right) \right). \tag{46}$$

6 Final model

Gathering together the contributions from all genotypes leads to the full model for evolution over multiple seasons as given in the main text, i.e.

$$x_{n+1} = x_{n+1}(x_n) + x_{n+1}(y_n) + x_{n+1}(z_n), \tag{47}$$

$$= \zeta_n \left(\epsilon_R \left(x_n + \frac{y_n}{4} \right) + \kappa_R \left(\beta_{RR} + \frac{\beta_{Rr}}{2} \right) \left(x_n + \frac{y_n}{2} \right) \right), \tag{48}$$

$$y_{n+1} = y_{n+1}(x_n) + y_{n+1}(y_n) + y_{n+1}(z_n), \tag{49}$$

$$= \zeta_n \left(\frac{\epsilon_R y_n}{2} + \kappa_R \left(\frac{\beta_{Rr}}{2} + \beta_{rr} \right) x_n + \frac{\kappa_R y_n}{2} + \kappa_r \left(\beta_{RR} + \frac{\beta_{Rr}}{2} \right) z_n \right), \tag{50}$$

$$z_{n+1} = z_{n+1}(x_n) + z_{n+1}(y_n) + z_{n+1}(z_n), \tag{51}$$

$$= \zeta_n \left(\frac{\epsilon_R y_n}{4} + \epsilon_r z_n + \left(\frac{\beta_{Rr}}{2} + \beta_{rr} \right) \left(\frac{\kappa_R y_n}{2} + \kappa_r z_n \right) \right). \tag{52}$$

In the above system

$$\begin{aligned}
\eta &= \frac{1}{1+(\nu-1)\alpha z_n}, && (53)\\
\beta_{RR} &= \eta x_n, && (54)\\
\beta_{Rr} &= \eta y_n, && (55)\\
\beta_{rr} &= \eta(1+(\nu-1)\alpha)z_n, && (56)\\
\omega_- &= 1-e^{-\gamma\eta}, && (57)\\
\omega_+ &= 1-e^{-\nu\gamma\eta}, && (58)\\
\theta_- &= (1-\omega_-)\sigma+\omega_-(1-\phi), && (59)\\
\mu_- &= \omega_-\phi, && (60)\\
\theta_+ &= (1-\omega_+)\sigma+\omega_+(1-\phi), && (61)\\
\mu_+ &= \omega_+\phi, && (62)\\
\epsilon_R &= \theta_-, && (63)\\
\kappa_R &= \mu_-, && (64)\\
\epsilon_r &= \alpha\delta\theta_+ + (1-\alpha)\theta_-, && (65)\\
\kappa_r &= \alpha\delta\mu_+ + (1-\alpha)\mu_-, && (66)
\end{aligned}$$

and ζ_n is chosen for each generation n to ensure $x_n + y_n + z_n = 1$.

References

[1] F. Chamchod and N.F. Britton. Analysis of a vector-bias model on malaria transmission. *Bulletin of Mathematical Biology*, 73:639–657, 2011.

[2] D.G. Lloyd. Some reproductive factors affecting the selection of self-fertilization in plants. *The American Naturalist*, 113:67–79, 1979.

[3] M.T. Morgan, W.G. Wilson, and T.M. Knight. Plant population dynamics, pollinator foraging, and the selection of self-fertilization. *The American Naturalist*, 166:169–183, 2005.
