# Supplementary material for: Virus Infection of Plants Alters Pollinator Preference: A Payback for Susceptible Hosts?
Source: PLoS Pathog. 2016 Aug 11;12(8):e1005790. doi: 10.1371/journal.ppat.1005790 (PMC4981420; doi:10.1371/journal.ppat.1005790)
Supplement: S1 Fig — CMV-Fny accumulates to a higher titer than CMVΔ2b in systemically-infected tomato leaves. (A) Semi-quantitative reverse transcription-polymerase chain reaction (RT-PCR) analysis of viral RNA (vRNA) accumulation leaves of tomato plants systemically infected with CMV-Fny (CMV) or CMVΔ2b. Leaf tissue samples were harvested for RNA extraction at 10 and 18 days post-inoculation (dpi). CMV RNA accumulation was determined by RT-PCR after 30 cycles of PCR and compared to the levels of the elongation factor 1 alpha (EF1α) transcript (to act as an internal loading control). The CMV-specific PCR products from CMV-infected leaves accumulated to higher levels than those from CMVΔ2b infected leaves. (B) RT-quantitative PCR of CMV accumulation relative to CMVΔ2b. Graph shows the mean accumulation of viral RNA in systemically-infected tissues of plants inoculated with CMV-Fny (CMV) or CMVΔ2b (Δ2b) at 10 and 18 dpi. Mean accumulation of virus-specific PCR products is shown for CMV and CMVΔ2b and error bars represent standard errors around the mean for n = 4 samples for CMVΔ2b at 10 dpi and n = 3 and 2, respectively, for CMV at 10 and 18dpi. The housekeeping transcript control was EF1α and levels are shown relative to CMVΔ2b, which is designated as ‘1’. (PDF) [file ppat.1005790.s004.pdf]

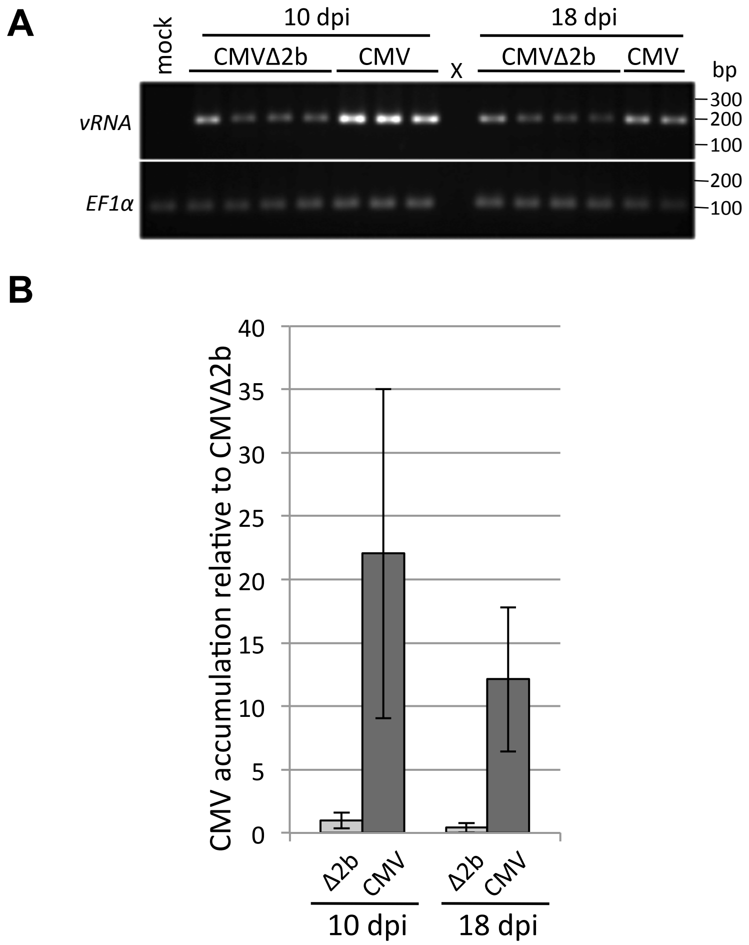

S1 Figure Detection and quantification of CMV-Fny and CMVΔ2b RNA in tomato CMV-Fny accumulates to a higher titer than CMVΔ2b in systemically-infected tomato leaves. (A) Semi-quantitative reverse transcription-polymerase chain reaction (RT-PCR) analysis of viral RNA (*vRNA*) accumulation leaves of tomato plants systemically infected with CMV-Fny (CMV) or CMVΔ2b. Leaf tissue samples were harvested for RNA extraction at 10 and 18 days post-inoculation (dpi). CMV RNA accumulation was determined by RT-PCR after 30 cycles of PCR and compared to the levels of the *elongation factor 1 alpha* (*EF1α*) transcript (to act as an internal loading control). The CMV-specific PCR products from CMV-infected leaves accumulated to higher levels than those from CMVΔ2b infected leaves. (B) RT-quantitative PCR of CMV accumulation relative to CMVΔ2b. Graph shows the mean accumulation of viral RNA in systemically-infected tissues of plants inoculated with CMV-Fny (CMV) or CMVΔ2b (Δ2b) at 10 and 18 dpi. Mean accumulation of virus-specific PCR products is shown for CMV and CMVΔ2b and error bars represent standard errors around the mean for n= 4 samples for CMVΔ2b at 10 dpi and n = 3 and 2, respectively, for CMV at 10 and 18dpi. The housekeeping transcript control was *EF1α* and levels are shown relative to CMVΔ2b, which is designated as ‘1’.
