# Supplementary material for: Virus Infection of Plants Alters Pollinator Preference: A Payback for Susceptible Hosts?
Source: PLoS Pathog. 2016 Aug 11;12(8):e1005790. doi: 10.1371/journal.ppat.1005790 (PMC4981420; doi:10.1371/journal.ppat.1005790)
Supplement: S2 Fig — (A) The tomato-cucumber mosaic virus (CMV) pathosystem is a virus-plant interaction in which infection with CMV-Fny does not greatly affect the mean number of flowers produced per plant in infected (CMV) versus mock-inoculated (Mock) tomato plants (n = 6 plants; one-way ANOVA: F(1,10) = 0.024, p = 0.8803). (B) Infection with CMV-Fny does not greatly affect the morphology of tomato flowers. Typical flowers from mock-inoculated (Mock) plants and plants infected with CMV-Fny (CMV) are shown. C. Infection with CMV-Fny stunts host growth. The mean height of plants at the point of flowering is shown for plants infected with CMV-Fny (CMV) versus mock-inoculated (Mock) plants (n = 4 plants and n = 3 plants, respectively; one-way ANOVA: F(1,5) = 52.92, p = 0.0077) (C). (D) CMV infection accelerated flowering; decreasing time to flowering (days post-sowing; n = 3 plants [CMV-Fny] and n = 4 plants [Mock]; one-way ANOVA: F(1,5) = 10.71, p = 0.0221). Mock, mock-inoculated; CMV, infected with CMV-Fny. Asterisks indicate significant differences. Error bars represent the standard error of the mean. (PDF) [file ppat.1005790.s005.pdf]

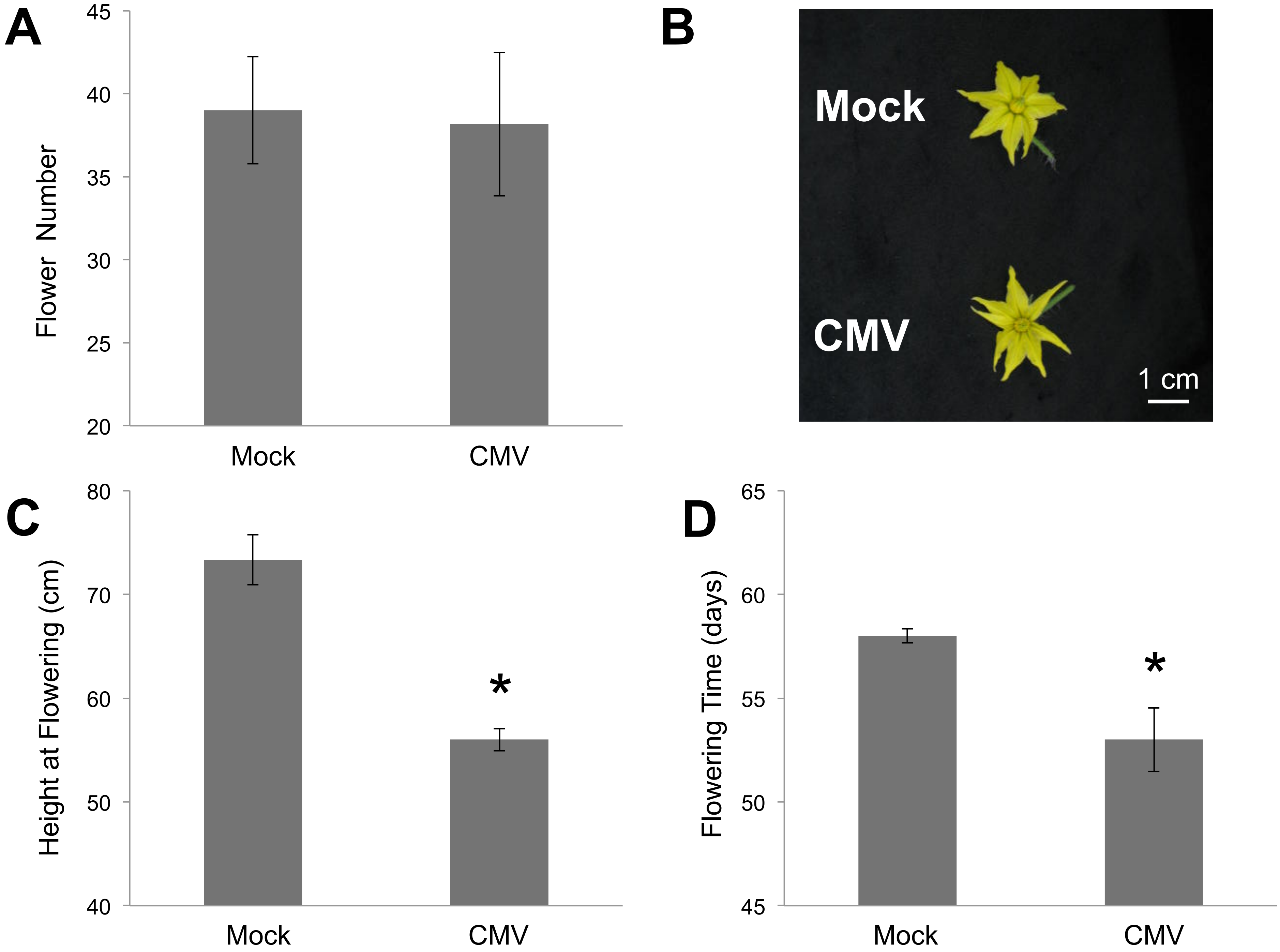

S2 Figure Impacts of CMV-Fny on tomato flowering characteristics A. The tomato-cucumber mosaic virus (CMV) pathosystem is a virus-plant interaction in which infection with CMV-Fny does not greatly affect the mean number of flowers produced per plant in infected (CMV) versus mock-inoculated (Mock) tomato plants (n = 6 plants; one-way ANOVA: $F(1,10) = 0.024$, $p = 0.8803$). B. Infection with CMV-Fny does not greatly affect the morphology of tomato flowers. Typical flowers from mock-inoculated (Mock) plants and plants infected with CMV-Fny (CMV) are shown. C. Infection with CMV-Fny stunts host growth. The mean height of plants at the point of flowering is shown for plants infected with CMV-Fny (CMV) versus mock-inoculated (Mock) plants (n = 4 plants and n = 3 plants, respectively; one-way ANOVA: $F(1,5) = 52.92$, $p = 0.0077$) (C). (D) CMV infection accelerated flowering; decreasing time to flowering (days post-sowing; n = 3 plants [CMV-Fny] and n = 4 plants [Mock]; one-way ANOVA: $F(1,5) = 10.71$, $p = 0.0221$). Mock, mock-inoculated; CMV, infected with CMV-Fny. Asterisks indicate significant differences. Error bars represent the standard error of the mean.
