# Supplementary material for: Virus Infection of Plants Alters Pollinator Preference: A Payback for Susceptible Hosts?
Source: PLoS Pathog. 2016 Aug 11;12(8):e1005790. doi: 10.1371/journal.ppat.1005790 (PMC4981420; doi:10.1371/journal.ppat.1005790)
Supplement: S3 Fig — (A) Pollen yield from mock-inoculated and virus infected flowers is similar. Fully open flowers from 12 mock-inoculated (mock) and nine CMV-PV0187-infected (CMV) plants were excised into microfuge tubes containing 300μl of water and vortexed for 40 seconds. Using a microscope, released pollen grains were counted in technical triplicates using a cell-counting chamber. The mean number of pollen grains released by flowers is shown. Error bars indicate standard error around the mean. (B) The viability of pollen from mock-inoculated and CMV-infected flowers is similar. Pollen was harvested into microfuge tubes from flowers (at developmental stage 6) by manual buzzing with an electrical toothbrush and stained with fluorescein diacetate. Data are from nine mock-inoculated and nine CMV-PV0187 infected plants. Esterase activity in viable pollen grains releases fluorescein that fluoresces under blue light (Li, X., 2011 http://www.bio-protocol.org/e75) [58] (see panel C). The percentage of pollen grains fluorescing (i.e. viable) is indicated with error bars indicating standard error around the mean. (C) Typical microscopic fields of view for pollen grains extracted from flowers of mock-inoculated (mock) and CMV-PV0187-infected (CMV) plants viewed under blue light and bright field with an epi-fluorescent microscope (DMRXA, Zeiss) connected to a digital camera (DFC425, Zeiss). Upper panels were viewed with blue light illumination under bright field optics enabling viable (fluorescent) and non-viable (non-fluorescent) pollen grains to be counted. Lower panels show pollen grains viewed with epi-fluorescent optics only. Scale bar = 100μm. (PDF) [file ppat.1005790.s006.pdf]

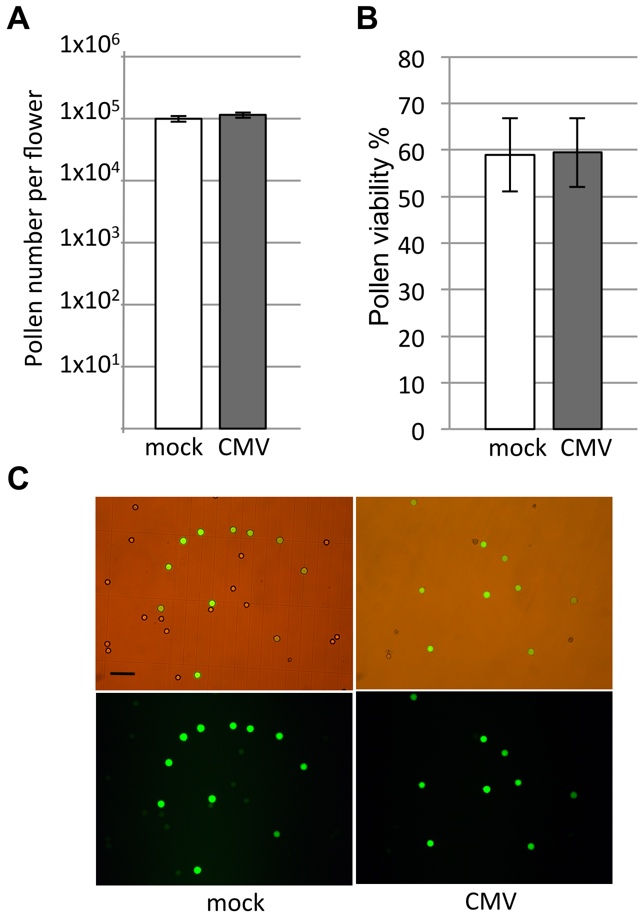

S3 Figure Characteristics of pollen from flowers of CMV-infected and mock-inoculated tomato plants (A) Pollen yield from mock-inoculated and virus infected flowers is similar. Fully open flowers from 12 mock-inoculated (mock) and nine CMV-PV0187-infected (CMV) plants were excised into microfuge tubes containing 300µl of water and vortexed for 40 seconds. Using a microscope, released pollen grains were counted in technical triplicates using a cell-counting chamber. The mean number of pollen grains released by flowers is shown. Error bars indicate standard error around the mean. (B) The viability of pollen from mock-inoculated and CMV-infected flowers is similar. Pollen was harvested into microfuge tubes from flowers (at developmental stage 6) by manual buzzing with an electrical toothbrush and stained with fluorescein diacetate. Data are from nine mock-inoculated and nine CMV-PV0187 infected plants. Esterase activity in viable pollen grains releases fluorescein that fluoresces under blue light (Li, X., 2011 http://www.bio-protocol.org/e75) [58] (see panel C). The percentage of pollen grains fluorescing (i.e. viable) is indicated with error bars indicating standard error around the mean. (C) Typical microscopic fields of view for pollen grains extracted from flowers of mock-inoculated (mock) and CMV-PV0187-infected (CMV) plants viewed under blue light and bright field with an epi-fluorescent microscope (DMRXA, Zeiss) connected to a digital camera (DFC425, Zeiss). Upper panels were viewed with blue light illumination under bright field optics enabling viable (fluorescent) and non-viable (non-fluorescent) pollen grains to be counted. Lower panels show pollen grains viewed with epi-fluorescent optics only. Scale bar = 100µm.
