# Supplementary material for: Virus Infection of Plants Alters Pollinator Preference: A Payback for Susceptible Hosts?
Source: PLoS Pathog. 2016 Aug 11;12(8):e1005790. doi: 10.1371/journal.ppat.1005790 (PMC4981420; doi:10.1371/journal.ppat.1005790)
Supplement: S4 Fig — The three genomic RNAs of CMV-PV0187 were sequenced. The RNA sequences were compared to those of CMV-Fny and other CMV strains and isolates. (A) Phylogenetic analysis using the RNA sequences of CMV-PV0187 RNAs 1, 2, and 3, with corresponding sequences of other CMV strains and isolates. Phylogenetic analysis using the neighbour-joining method under the Kimura-2 parameter was conducted in MEGA software (Version 6.06). The bootstrap consensus tree was carried out with 1000 replications. Panels (left to right) show the phylogenetic analysis of RNAs1, 2 and 3. The CMV-PV0187 sequence data used in this analysis is available at NCBI under GenBank accession numbers KP165580, KP165581 and KP165582 corresponding to RNA1, RNA2, and RNA3, respectively. PV0187-CMV groups closely with CMV-Fny (indicated with red diamonds), with which it has an overall 99% RNA sequence identity. (B) The predicted 110 residue amino acid sequences of the 2b proteins of CMV-Fny (Fny 2b: upper sequence) and CMV-PV0187 (PV0187 2b: lower sequence) are identical. The amino acid sequences are a virtual translation of the 2b open reading frames of the two CMV strains. The numbers 60, 61, and 110 indicate amino acid residue positions. (PDF) [file ppat.1005790.s007.pdf]

A

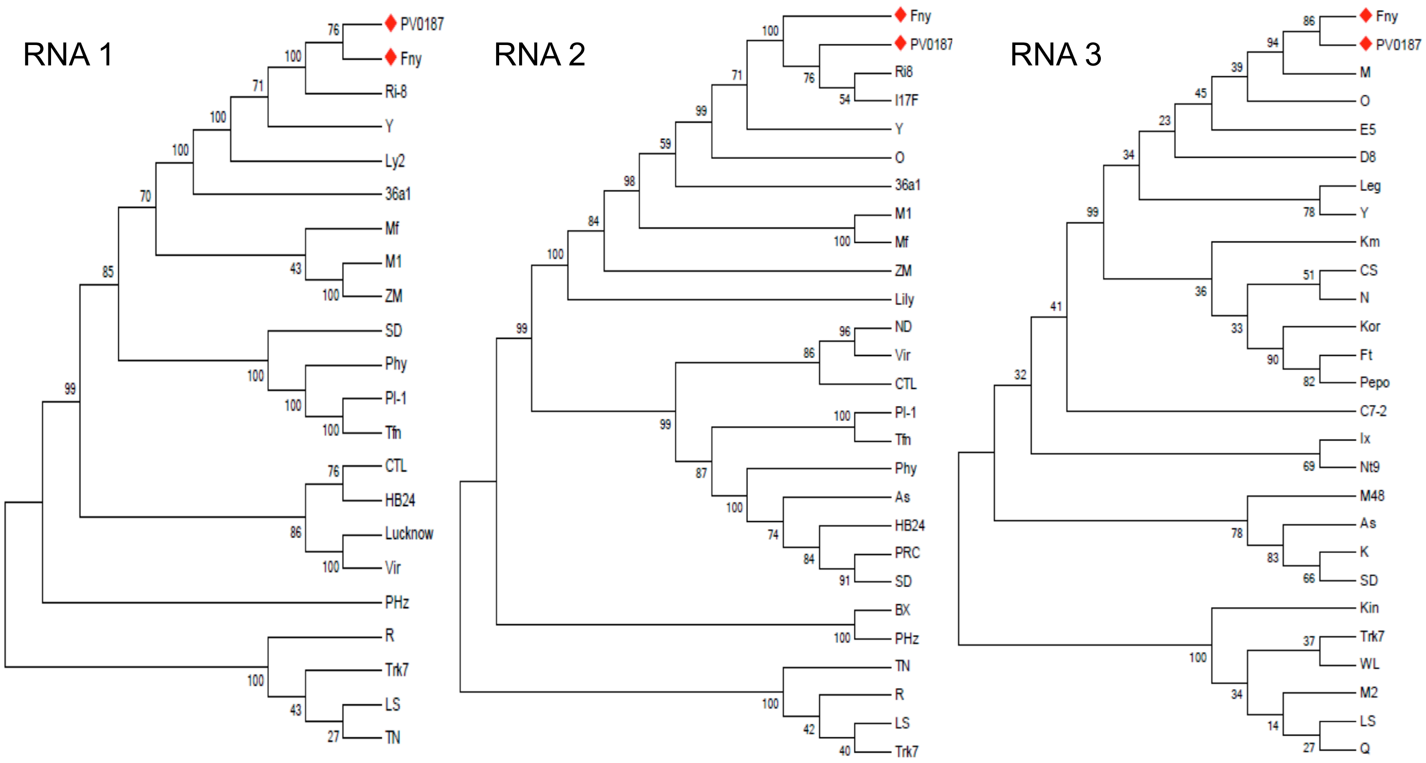

B

Fny 2b MELNVGAMTNVELQLARMVEAKKQRRRSHKQNRRERGHSPSERARSNLRLFRFLPFYQV 60

PV0187 2b MELNVGAMTNVELQLARMVEAKKQRRRSHKQNRRERGHSPSERARSNLRLFRFLPFYQV 60

Fny 2b 61 DGSELTGSCRHVNVAELPESEASRLELSAEDHDFDDTDWFAGNEWAEGAF 110

PV0187 2b 61 DGSELTGSCRHVNVAELPESEASRLELSAEDHDFDDTDWFAGNEWAEGAF 110

S4 Figure Sequencing and phylogenetic analysis of CMV-PV0187 The three genomic RNAs of CMV-PV0187 were sequenced. The RNA sequences were compared to those of CMV-Fny and other CMV strains and isolates. (A) Phylogenetic analysis using the RNA sequences of CMV-PV0187 RNAs 1, 2, and 3, with corresponding sequences of other CMV strains and isolates. Phylogenetic analysis using the neighbour-joining method under the Kimura-2 parameter was conducted in MEGA software (Version 6.06). The bootstrap consensus tree was carried out with 1000 replications. Panels (left to right) show the phylogenetic analysis of RNAs1, 2 and 3. The CMV-PV0187 sequence data used in this analysis is available at NCBI under GenBank accession numbers KP165580, KP165581 and KP165582 corresponding to RNA1, RNA2, and RNA3, respectively. PV0187-CMV groups closely with CMV-Fny (indicated with red diamonds), with which it has an overall 99% RNA sequence identity. (B) The predicted 110 residue amino acid sequences of the 2b proteins of CMV-Fny (Fny 2b: upper sequence) and CMV-PV0187 (PV0187 2b: lower sequence) are identical. The amino acid sequences are a virtual translation of the 2b open reading frames of the two CMV strains. The numbers 60, 61, and 110 indicate amino acid residue positions.
