# Supplementary material for: Virus Infection of Plants Alters Pollinator Preference: A Payback for Susceptible Hosts?
Source: PLoS Pathog. 2016 Aug 11;12(8):e1005790. doi: 10.1371/journal.ppat.1005790 (PMC4981420; doi:10.1371/journal.ppat.1005790)
Supplement: S5 Fig — The growth and morphology of leaves, flowers and fruit were compared between tomato plants that had been mock-inoculated or infected with CMV-PV0187. Plants or plant organs were photographed and typical images are shown in panels A-E. (A) Tomato plants inoculated with CMV-PV0187 at the seedling stage show marked stunting (right) compared to mock-inoculated plants (left). (B) Mature, expanded leaves of infected (left) and mock-inoculated (right) plants. (C) Young, upper leaves of infected (left) and mock-inoculated (right) plants. (D) Flowers from mock-inoculated (left) and CMV-PV0187 (right) infected plants are similar in appearance and show no gross differences in morphology. (E) Tomato fruits from mock-inoculated plants (left) are larger than those from CMV-PV0187 infected (right) plants. Scale bars = 3 cm. (PDF) [file ppat.1005790.s008.pdf]

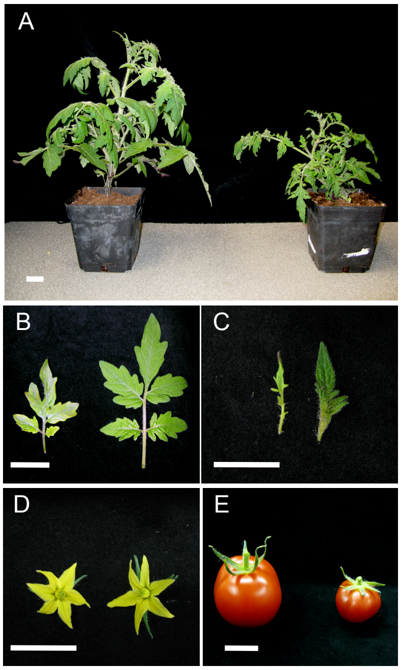

S5 Figure Impact of CMV-PV0187 on tomato plants The growth and morphology of leaves, flowers and fruit were compared between tomato plants that had been mock-inoculated or infected with CMV-PV0187. Plants or plant organs were photographed and typical images are shown in panels A-E. (A) Tomato plants inoculated with CMV-PV0187 at the seedling stage show marked stunting (right) compared to mock-inoculated plants (left). (B) Mature, expanded leaves of infected (left) and mock-inoculated (right) plants. (C) Young, upper leaves of infected (left) and mock-inoculated (right) plants. (D) Flowers from mock-inoculated (left) and CMV-PV0187 (right) infected plants are similar in appearance and show no gross differences in morphology. (E) Tomato fruits from mock-inoculated plants (left) are larger than those from CMV-PV0187 infected (right) plants. Scale bars = 3 cm.
