# Supplementary material for: Virus Infection of Plants Alters Pollinator Preference: A Payback for Susceptible Hosts?
Source: PLoS Pathog. 2016 Aug 11;12(8):e1005790. doi: 10.1371/journal.ppat.1005790 (PMC4981420; doi:10.1371/journal.ppat.1005790)
Supplement: S6 Fig — Bees spent longer buzz-pollinating (sonicating) flowers on CMV-infected tomato plants. Single bees were released into the flight arena containing three mock-inoculated and three CMV-infected flowering tomato plants (Fig 6; S8 Fig). The time each bee spent buzz-pollinating its first five choices of flower was measured using a stopwatch. n = the number of bees making each choice. (PDF) [file ppat.1005790.s009.pdf]

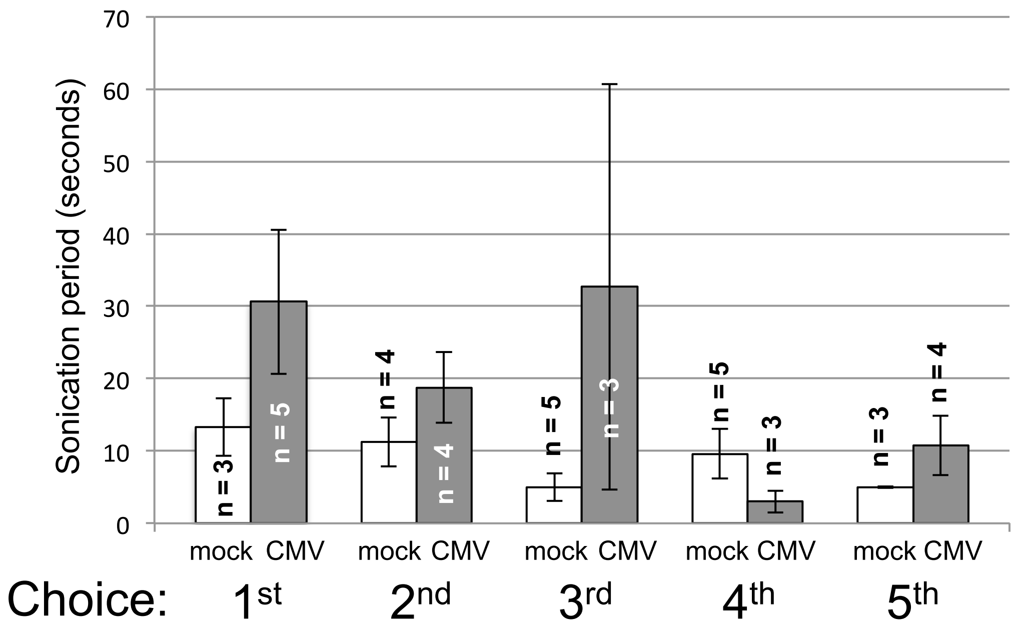

S6 Figure Choices and timings for buzz pollination by bumblebees Bees spent longer buzz-pollinating (sonicating) flowers on CMV-infected tomato plants. Single bees were released into the flight arena containing three mock-inoculated and three CMV-infected flowering tomato plants (Fig 6; S8 Figure). The time each bee spent buzz-pollinating its first five choices of flower was measured using a stopwatch. n= the number of bees making each choice.
