# Supplementary material for: Virus Infection of Plants Alters Pollinator Preference: A Payback for Susceptible Hosts?
Source: PLoS Pathog. 2016 Aug 11;12(8):e1005790. doi: 10.1371/journal.ppat.1005790 (PMC4981420; doi:10.1371/journal.ppat.1005790)
Supplement: S7 Fig — (A) Growth rate of resistant mutants in the vicinity of the (0,0,1) equilibrium at which only susceptible plants are present. The panel shows a series of full two-way sensitivity analyses of the model, showing effects on the growth rate of rare mutant resistant plants in the vicinity of the equilibrium at which only susceptible plants are present, caused by independently changing pairs of parameters (all other parameters fixed). All pair-wise combinations of two parameters are shown: dots on each axis show default values of each parameter. In all cases, the magnitude of the largest Eigenvalue of the Jacobian matrix at the model equilibrium–which is equivalent to the initial discrete time rate of exponential growth over successive seasons of rare mutant resistant plants -is shown by color. Note that Fig 8 in the main text characterises long-term evolutionary outcomes by distinguishing regions in which growth rates of each type of mutant are larger than or smaller than one, and so in which the equilibria can be invaded (or not): these results therefore provide additional numerical detail in support of that figure. (B) Growth rate of susceptible mutant plants in the vicinity of the (1,0,0) equilibrium at which only homozygous resistant plants are present (all other details as per Panel A). (PDF) [file ppat.1005790.s010.pdf]

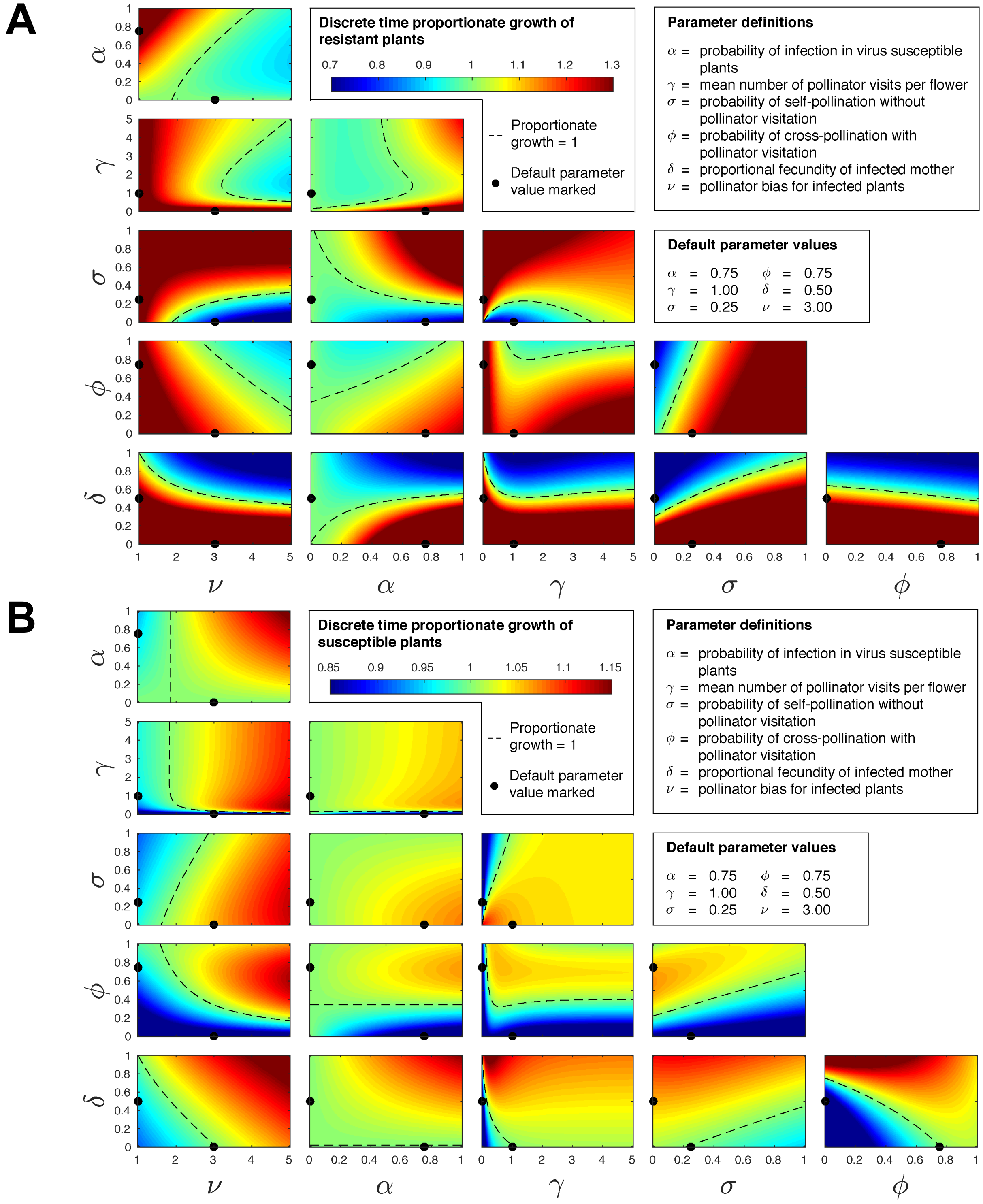

S7 Figure. Sensitivity analysis showing discrete time exponential growth rates for a rare mutant type of plant in the vicinity of the equilibrium from which that type is absent. (A) Growth rate of resistant mutants in the vicinity of the (0,0,1) equilibrium at which only susceptible plants are present. The panel shows a series of full two-way sensitivity analyses of the model, showing effects on the growth rate of rare mutant resistant plants in the vicinity of the equilibrium at which only susceptible plants are present, caused by independently changing pairs of parameters (all other parameters fixed). All pair-wise combinations of two parameters are shown: dots on each axis show default values of each parameter. In all cases, the magnitude of the largest Eigenvalue of the Jacobian matrix at the model equilibrium – which is equivalent to the initial discrete time rate of exponential growth over successive seasons of rare mutant resistant plants – is shown by color. Note that Figure 8 in the main text characterises long-term evolutionary outcomes by distinguishing regions in which growth rates of each type of mutant are larger than or smaller than one, and so in which the equilibria can be invaded (or not): these results therefore provide additional numerical detail in support of that figure. (B) Growth rate of susceptible mutant plants in the vicinity of the (1,0,0) equilibrium at which only homozygous resistant plants are present (all other details as per Panel A).
