# Supplementary material for: Virus Infection of Plants Alters Pollinator Preference: A Payback for Susceptible Hosts?
Source: PLoS Pathog. 2016 Aug 11;12(8):e1005790. doi: 10.1371/journal.ppat.1005790 (PMC4981420; doi:10.1371/journal.ppat.1005790)
Supplement: S8 Fig — Design of free choice bee-pollination experiment. (A) A large flight arena (125 x 370 x 90cm: H x W x D) was constructed out of nylon netting with three zipped doors to allow full access. Within this flight arena (zone delineated with a white rectangle) a bumblebee colony was attached by a tube to a small flight arena (38 x 44 x 71 cm; H x W x D) containing a microtiter plate filled with 30% sucrose (not visible) to allow the bumblebees to feed freely. Sliding gates on the side of the small arena permitted one bee to be released into the larger arena containing three mock-inoculated and three cucumber mosaic virus (CMV)-infected flowering tomato plants. (B) Cartoon demonstrating the arrangement of mock-inoculated (green plants) and CMV-infected plants (red) within the larger flight arena. (PDF) [file ppat.1005790.s011.pdf]

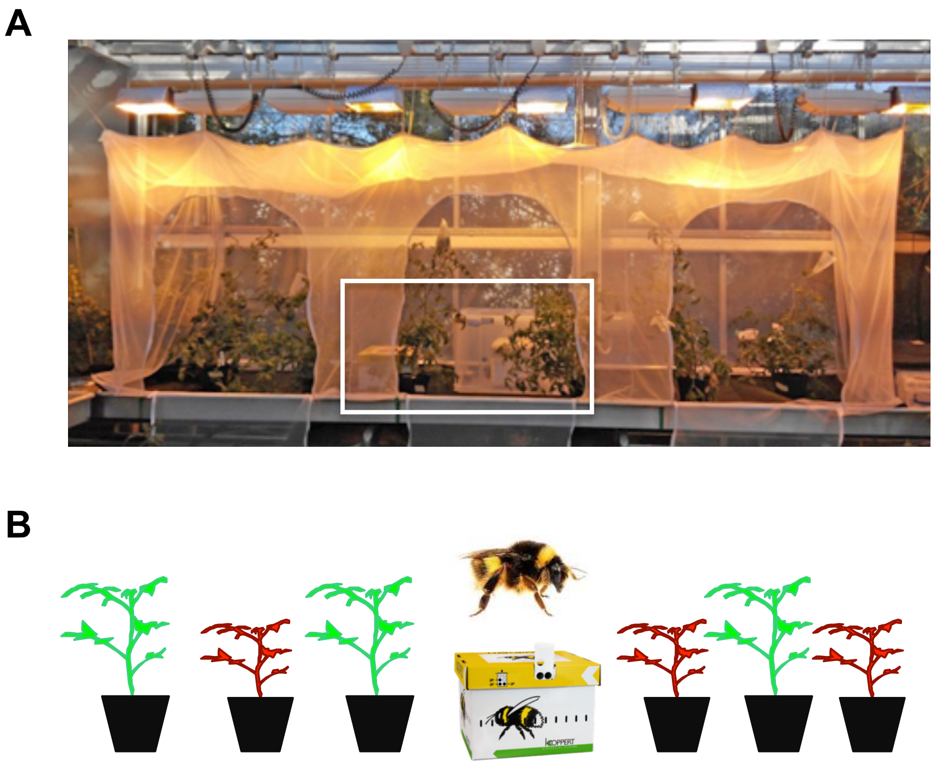

S8 Figure Arena used for pollination experiments Design of free choice bee-pollination experiment. (A) A large flight arena (125 x 370 x 90cm: H x W x D) was constructed out of nylon netting with three zipped doors to allow full access. Within this flight arena (zone delineated with a white rectangle) a bumblebee colony was attached by a tube to a small flight arena (38 x 44 x 71 cm; H x W x D) containing a microtiter plate filled with 30% sucrose (not visible) to allow the bumblebees to feed freely. Sliding gates on the side of the small arena permitted one bee to be released into the larger arena containing three mock-inoculated and three cucumber mosaic virus (CMV)-infected flowering tomato plants. (B) Cartoon demonstrating the arrangement of mock-inoculated (green plants) and CMV-infected plants (red) within the larger flight arena.
