# Supplementary material for: Correction: Virus Infection of Plants Alters Pollinator Preference: A Payback for Susceptible Hosts?
Source: PLoS Pathog. 2016 Sep 15;12(9):e1005906. doi: 10.1371/journal.ppat.1005906 (PMC5025089; doi:10.1371/journal.ppat.1005906)
Supplement: S2 File — (PDF) [file ppat.1005906.s002.PDF]

PLOS | PATHOGENS

RESEARCH ARTICLE

Virus Infection of Plants Alters Pollinator Preference: A Payback for Susceptible Hosts?

Simon C. Groen[1☯¤a], Sanjie Jiang[1☯], Alex M. Murphy[1☯], Nik J. Cunniffe[1☯], Jack H. Westwood[1☯¤b], Matthew P. Davey[1], Toby J. A. Bruce[2], John C. Caulfield[2], Oliver J. Furzer[1¤c], Alison Reed[1], Sophie I. Robinson[1], Elizabeth Miller[1], Christopher N. Davis[1¤d], John A. Pickett[2], Heather M. Whitney[3], Beverley J. Glover[1], John P. Carr[1]*

1 Department of Plant Sciences, University of Cambridge, Cambridge, United Kingdom, **2** Rothamsted Research, Harpenden, Hertfordshire, United Kingdom, **3** University of Bristol, School of Biological Sciences, Bristol, United Kingdom

☯ These authors contributed equally to this work.
¤a Current address: Department of Biology, Center for Genomics and Systems Biology, New York University, New York, New York, United States of America
¤b Current address: 2Blades Foundation, Evanston, Illinois, United States of America
¤c Current address: The Sainsbury Laboratory, Norwich Research Park, Norwich, United Kingdom
¤d Current address: Centre for Complexity Science, Zeeman Building, University of Warwick, Coventry, United Kingdom
* jpc1005@hermes.cam.ac.uk

OPEN ACCESS

Citation: Groen SC, Jiang S, Murphy AM, Cunniffe NJ, Westwood JH, Davey MP, et al. (2016) Virus Infection of Plants Alters Pollinator Preference: A Payback for Susceptible Hosts? PLoS Pathog 12(8): e1005790. doi:10.1371/journal.ppat.1005790

Editor: Anna Whitfield, Kansas State University, UNITED STATES

Received: November 27, 2015

Accepted: July 6, 2016

Published: August 11, 2016

Copyright: © 2016 Groen et al. This is an open access article distributed under the terms of the Creative Commons Attribution License, which permits unrestricted use, distribution, and reproduction in any medium, provided the original author and source are credited.

Data Availability Statement: All relevant data are within the paper and its Supporting Information files except for the sequences of RNAs 1, 2 and 3 of CMV isolate PV0187 which are available from GenBank (http://www.ncbi.nlm.nih.gov/genbank/) under accession numbers KP165580, KP165581, and KP165582, respectively.

Funding: Major funding for this project was provided to JPC by the Leverhulme Trust (Grant numbers RPG-2012-667 and F/09741/F: https://www.leverhulme.ac.uk/). Additional funding to JPC and studentships to support JHW and SCG came from the Biotechnological and Biological Sciences

Abstract

Plant volatiles play important roles in attraction of certain pollinators and in host location by herbivorous insects. Virus infection induces changes in plant volatile emission profiles, and this can make plants more attractive to insect herbivores, such as aphids, that act as viral vectors. However, it is unknown if virus-induced alterations in volatile production affect plant-pollinator interactions. We found that volatiles emitted by cucumber mosaic virus (CMV)-infected tomato (*Solanum lycopersicum)* and *Arabidopsis thaliana* plants altered the foraging behaviour of bumblebees (*Bombus terrestris*). Virus-induced quantitative and qualitative changes in blends of volatile organic compounds emitted by tomato plants were identified by gas chromatography-coupled mass spectrometry. Experiments with a CMV mutant unable to express the 2b RNA silencing suppressor protein and with Arabidopsis silencing mutants implicate microRNAs in regulating emission of pollinator-perceivable volatiles. In tomato, CMV infection made plants emit volatiles attractive to bumblebees. Bumblebees pollinate tomato by 'buzzing' (sonicating) the flowers, which releases pollen and enhances self-fertilization and seed production as well as pollen export. Without buzz-pollination, CMV infection decreased seed yield, but when flowers of mock-inoculated and CMV-infected plants were buzz-pollinated, the increased seed yield for CMV-infected plants was similar to that for mock-inoculated plants. Increased pollinator preference can potentially increase plant reproductive success in two ways: i) as female parents, by increasing the probability that ovules are fertilized; ii) as male parents, by increasing pollen export. Mathematical modeling suggested that over a wide range of conditions in the wild, these increases to the number of offspring of infected susceptible plants resulting from increased pollinator preference could outweigh underlying strong selection pressures favoring

Research Council (Grant number BB/J011762/1: http://www.bbsrc.ac.uk/). Other additional funding was obtained from the Isaac Newton Trust (http://www.newtontrust.cam.ac.uk/: grant number 12.07/I to AMM). The funders had no role in study design, data collection and analysis, decision to publish, or preparation of the manuscript.

Competing Interests: The authors have declared that no competing interests exist.

pathogen resistance, allowing genes for disease susceptibility to persist in plant populations. We speculate that enhanced pollinator service for infected individuals in wild plant populations might provide mutual benefits to the virus and its susceptible hosts.

Author Summary

Cucumber mosaic virus, an important pathogen of tomato, causes plants to emit volatile chemicals that attract bumblebees. Bumblebees are important tomato pollinators, but do not transmit this virus. We propose that under natural conditions, helping host reproduction by encouraging bee visitation might represent a 'payback' by the virus to susceptible hosts. Although tomato flowers can give rise to seed through self-fertilization, bumblebee-mediated 'buzz-pollination' enhances this, increasing the number of seeds produced per fruit. Buzz-pollination further favors reproductive success of a plant by facilitating pollen export. Mathematical modeling suggests that if self-fertilization by infected plants, as well as pollen transfer from these plants (cross-fertilization) to surrounding plants is increased, this might favor reproduction of susceptible over that of resistant plants. This raises the possibility that under natural conditions some viruses might enhance competitiveness of susceptible plants and inhibit the emergence of resistant plant strains. We speculate that it may be in a virus' interest to pay back a susceptible host by enhancing its attractiveness to pollinators, which will likely increase fertilization rates and the dissemination of susceptible plant pollen and may compensate for a decreased yield of seeds on the virus-infected plants.

Introduction

Insects pollinate many plant species, including several major crops [1]. Bees are the single most important insect pollinator group and can be a limiting factor for the success of plant reproduction [1–3]. Consequently, there is strong inter- and intra-specific competition among plants for the attention of pollinators [2, 3]. With respect to insect-pollinated crops, pollinator visitation (or artificial pollination) is required to obtain maximal seed and fruit production [4, 5]. Consequently, pollination facilitates higher yields even when a crop plant is self-compatible [4, 5]. Tomato (*Solanum lycopersicum*) provides a good example of the relationship between pollination and yield. Bumblebees are important pollinators of tomato and other *Solanum* species that utilize an unusual pollination system called 'buzz-pollination' [6]. Buzz-pollinated flowers provide excess pollen as a reward to foraging bumblebees that feed it to their developing larvae [6]. Although domesticated tomato is to a large extent 'self-fertilizing', buzz-pollination by bumblebees or by manual application of mechanical vibration 'wands' is required for maximal seed production, which in turn promotes increased fruit yield (see [5] and references therein).

Cucumber mosaic virus (CMV), one of the major viral pathogens of tomato, is a positive-sense RNA virus that encodes five proteins including the 2b protein, which is a viral suppressor of RNA silencing (VSR) [7, 8]. Bees do not transmit CMV but the virus is vectored by several aphid species [7, 8]. Virus infection causes dramatic changes in plant host metabolism (reviewed in [9]). CMV-induced metabolic changes include qualitative and quantitative alterations in the emission of volatile compounds and in certain host species this makes infected hosts more attractive to aphid vectors [10, 11].

It is not known if the virus-induced alterations in host volatile emission that influence aphid behavior can also affect plant-pollinator interactions. Most bee-plant interaction studies have focussed on the effects of visual cues. Therefore, the influences of floral and non-floral volatiles on bee-mediated pollination are less well understood [12–14]. In contrast, the floral odors that attract moth pollinators have been more extensively researched [15–17]. In this study we determined that CMV infection induced changes in olfactory cues emitted by *Arabidopsis thaliana* (hereafter referred to as Arabidopsis) and tomato plants in ways that could be perceived by the bumblebee *Bombus terrestris*, and confirmed in tomato that this was associated with quantitative and qualitative changes in the blend of plant-emitted volatile organic compounds (VOCs). We also elucidated a role for the host microRNA (miRNA) pathway in regulating the emission of bee-perceivable olfactory cues. Our data indicated that bumblebees possess an innate preference for olfactory signals emitted by CMV-infected tomato plants and we mathematically modeled what the possible wider implications of this might be if a similar preference occurred in wild host plants under natural conditions.

Results

Bumblebees Showed an Innate Preference for Volatiles Emitted by CMV-Infected Tomato Plants

In 'free-choice' assays, bumblebees encountered flight arenas containing ten tomato plants (five plants/treatment) concealed within towers designed to allow odors to diffuse out but prevent the bees from seeing or touching the plants (Fig 1A). Cups that were placed on top of towers hiding plants of both treatment groups offered bumblebees the identical 'incentive' of a 30% sucrose solution. Nonetheless, when presented with mock-inoculated and CMV-infected tomato plants, bumblebees preferred to visit the towers that were hiding infected plants (Fig 1B) (S1 Table). Bumblebees showed similar preferences for flowering and non-flowering CMV-infected plants, indicating that leaves were the main source of attractive volatiles (Fig 1B). Bumblebees also displayed a preference for CMV-infected tomato plants over plants infected with CMVΔ2b, a viral mutant lacking the gene for the 2b VSR (Fig 1B), a factor that also influences CMV-plant-aphid interactions [18,19].

Bumblebees Could Learn to Distinguish between Volatiles Emitted by CMV-Infected, Mutant and *2b*-Transgenic Arabidopsis Plants

The results obtained in free-choice assays with tomato plants infected with CMVΔ2b suggested that the 2b protein, which is a VSR, may be exerting effects on the metabolism of plant volatiles by interfering with host small RNA pathways. The model plant Arabidopsis is the best higher plant system to use to investigate the effects of small RNA pathways. However, whilst Arabidopsis plants emit potentially pollinator-influencing volatiles, this species is not bee-pollinated [20]. Consistent with this, bumblebees showed no significant difference in preference for volatiles emitted by CMV-infected *versus* mock-inoculated Arabidopsis plants in free-choice assays (Fig 1B).

An alternative approach to investigate the ability of bees to recognise differences in olfactory or other stimuli is to set up a differential conditioning or 'learning curve' assay [14,21]. A differential conditioning assay can reveal whether bees can perceive cues that would not normally induce any behavioural responses and that could not be studied in free-choice assays. In our differential conditioning assays, cups on towers offered bumblebees either a 30% sucrose solution 'reward' for choosing one treatment group or a 'punishment' (0.12% quinine) for choosing the other group [14,21]. Bumblebees cannot distinguish quinine from sucrose except by taste

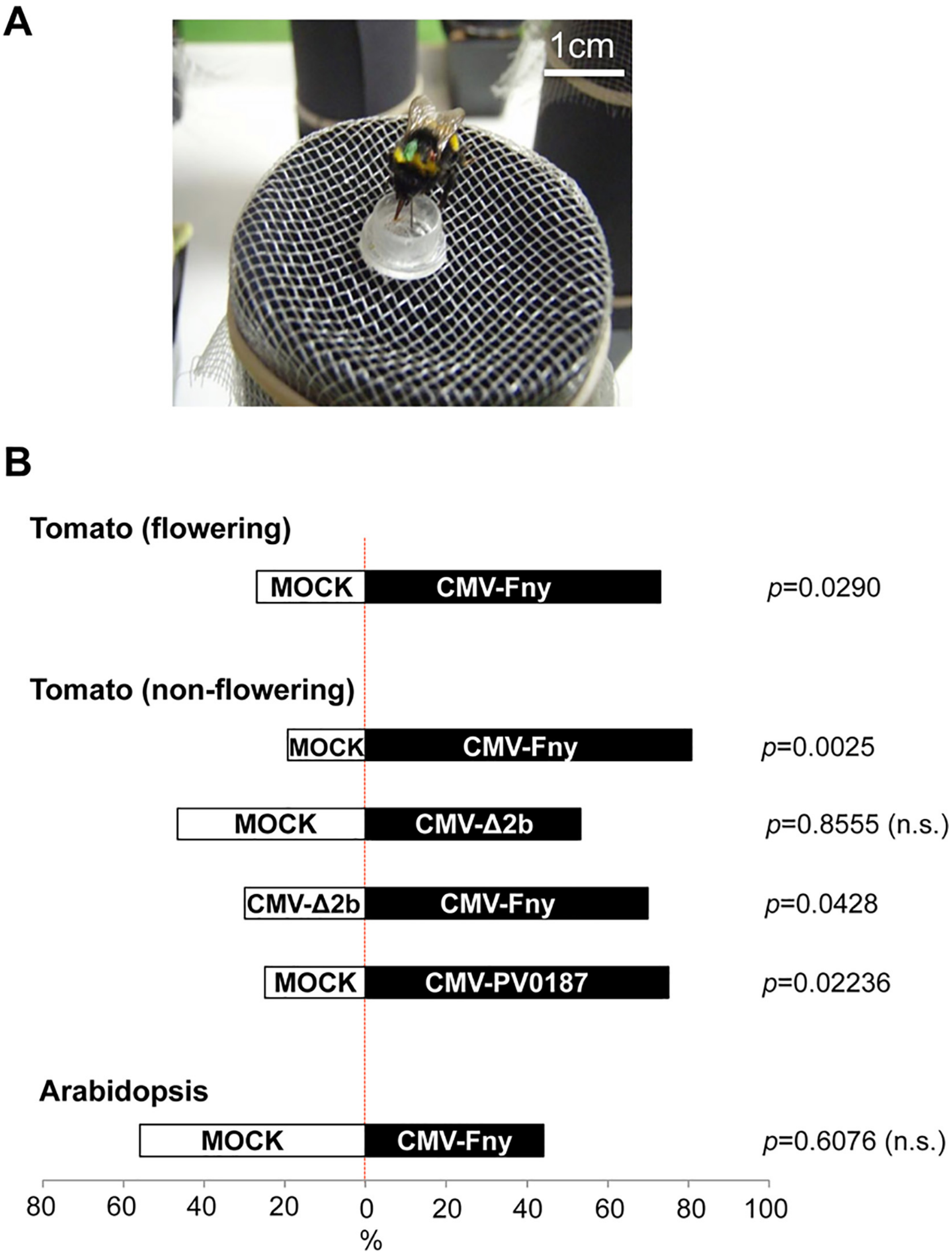

Fig 1. Bumblebees prefer volatiles emitted by CMV-infected tomato plants. (A) Experimental set-up: a bumblebee feeds from a sucrose-providing cup on a container placed over a test plant. (B) In free-choice assays, the volatiles induced by CMV infection bias visitation of bees toward CMV-infected over mock-inoculated tomato plants. This is shown for flowering plants (upper panel; n = 26) infected with CMV strain Fny (CMV-Fny) and non-flowering plants (middle panel) infected with CMV-Fny (n = 26) or CMV isolate PV0187 (PV0187) (n = 24). However, bumblebees showed no preference for plants infected with a viral mutant (derived from CMV-Fny) unable to express the 2b RNA silencing suppressor protein (CMVΔ2b; *n* = 30 for mock vs. CMVΔ2b; *n* = 30 for CMVΔ2b vs. CMV). Bumblebees showed no difference in preference for Arabidopsis plants that had been mock-inoculated or infected with CMV-Fny (*n* = 34) (significant differences indicated; n.s., non-significant: binomial test).

doi:10.1371/journal.ppat.1005790.g001

[22]. Thus, increasing frequency of visits to sucrose-offering towers over the course of an experiment indicated that bees have learned to use plant odor as a cue to identify and avoid drinking from cups placed on towers offering quinine solutions. In these assays, a steep learning curve shows that bumblebees can easily distinguish between two treatment groups, and indicates that the volatile blends are likely to be qualitatively and/or quantitatively very distinct, whereas less steep curves indicate that differences between blends are less marked, and that bees find it more difficult to learn to distinguish between them based on odor. An illustration of the power of this approach is shown in Fig 2 (S2 Table). Although bumblebees displayed an innate preference for volatiles emitted by CMV-infected tomato plants in free choice assays (Fig 1A), they could be trained by differential conditioning to overcome their innate preference and instead preferentially visit mock-inoculated tomato plants and avoid CMV-infected plants (Fig 2A).

Although we had observed that bumblebees had no innate preference for, or aversion to, volatiles emitted by Arabidopsis plants (Fig 1B), differential conditioning assays revealed that the insects could recognize differences between volatiles emitted by Arabidopsis plants that had been mock-inoculated and by plants that were infected with CMV (Fig 3A) (S2 Table). Bumblebees could also distinguish between CMV-infected and CMVΔ2b-infected Arabidopsis plants (Fig 3B). Hence, although they exhibit no innate behavioural response to the volatile blends emitted by Arabidopsis plants, differential conditioning assays showed that bumblebees could perceive differences in volatiles emitted by these plants. This meant that differential conditioning assays could permit further dissection of the mechanisms underlying CMV-induced changes in volatile emission using Arabidopsis as a model system.

Bumblebees could learn to differentiate transgenic plants constitutively expressing the 2b VSR from non-transgenic plants (Fig 3C) and from control-transgenic plants that were expressing an untranslatable *2b* transcript (Fig 3D). However, the insects displayed less ability to learn to distinguish mock-inoculated from CMVΔ2b-infected plants (Fig 3E). Comparison of the learning curves in Fig 3A *versus* Fig 3E by logistic regression (see Methods) indicated that bumblebees were better at distinguishing mock-inoculated plants from CMV-infected plants than from CMVΔ2b-infected plants ($\chi^2(1) = 40.17$, $p < 0.0001$). Bees could not be trained to differentiate non-transgenic plants from control-transgenic plants expressing a non-translatable *2b* transcript (Fig 3F).

The results with CMVΔ2b suggested that the 2b VSR plays an important role in altering the emission of bee-perceivable olfactory cues emitted by tomato and Arabidopsis plants (Figs 1A and 3E). However, CMVΔ2b accumulates to lower levels in plants than wild-type CMV and in previous work it was found that viral titer, as well as the presence of the 2b protein, plays a role in modification of the interactions of Arabidopsis with aphids [19]. Hence, it was conceivable that differences in virus titer might affect the emission of bee-perceivable volatiles by plants infected by CMV or CMVΔ2b and explain why the bees found it difficult to distinguish CMVΔ2b-infected plants from mock-inoculated plants. However, it is known that CMVΔ2b accumulates to levels comparable to those of wild type CMV in Arabidopsis plants carrying mutations in the genes encoding the Dicer-like (DCL) endoribonucleases DCL2 and DCL4, which are important factors in antiviral silencing [19]. Therefore, we examined the ability of bumblebees to learn to distinguish between volatile blends emitted by CMVΔ2b-infected and mock-inoculated *dcl2/4* double mutant plants (Fig 3G). The resulting learning curve (Fig 3G) was not significantly different from that obtained using wild-type plants that had been mock-inoculated or infected with CMVΔ2b (Fig 3E) ($\chi^2(1) = 0.66$, $p = 0.42$), indicating that an increase in CMVΔ2b titer did not enhance bee learning. Although we cannot rule out a role for other CMV gene products, the results indicate that the 2b VSR is the most significant viral factor conditioning changes in the emission of bee-perceivable volatiles.

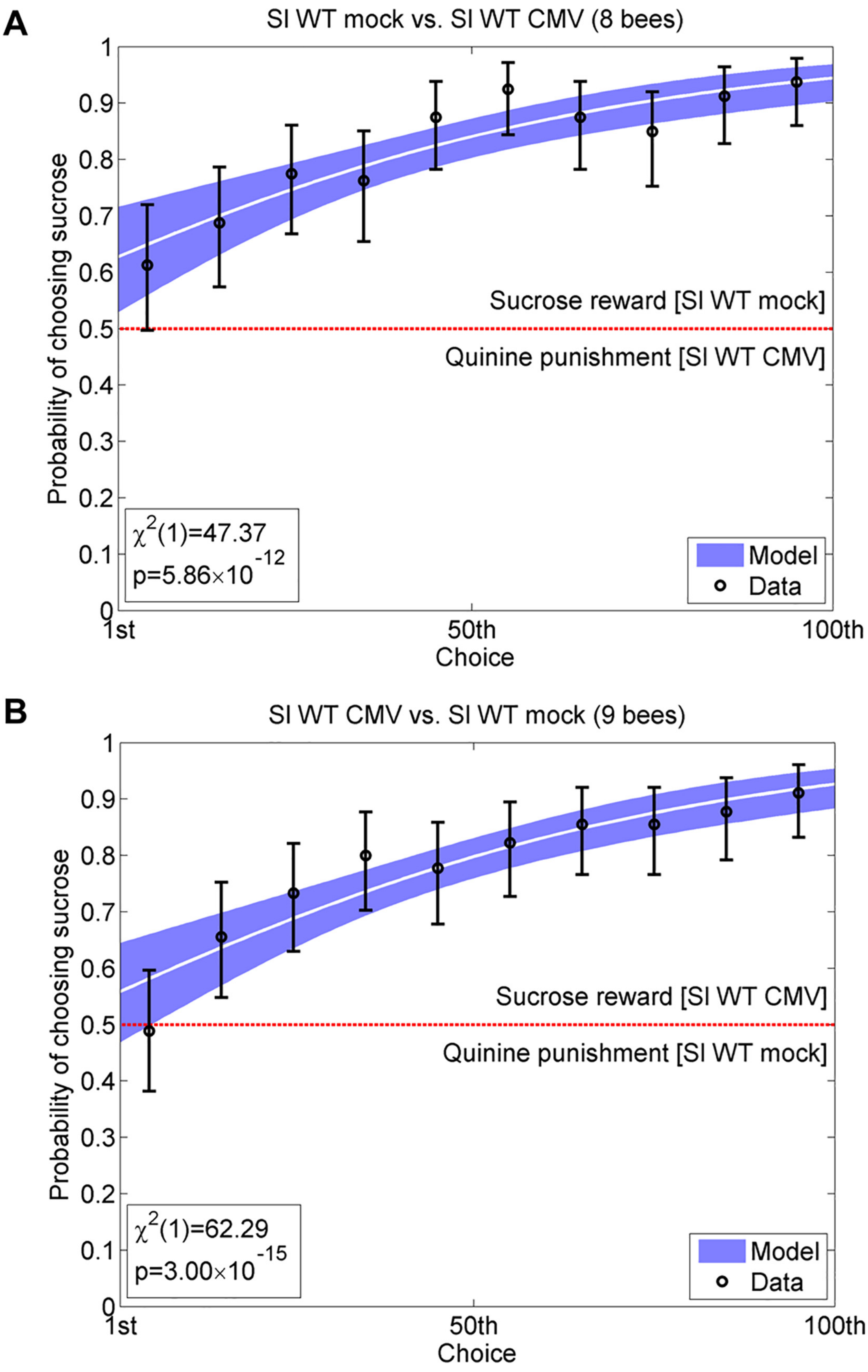
A
Sl WT mock vs. Sl WT CMV (8 bees)
Probability of choosing sucrose
Sucrose reward [Sl WT mock]
Quinine punishment [Sl WT CMV]
χ²(1)=47.37
p=5.86×10^-12
Model
Data
1st
50th
100th
Choice
B
Sl WT CMV vs. Sl WT mock (9 bees)
Probability of choosing sucrose
Sucrose reward [Sl WT CMV]
Quinine punishment [Sl WT mock]
χ²(1)=62.29
p=3.00×10^-15
Model
Data
1st
50th
100th
Choice

Fig 2. Bumblebees can be conditioned to disregard their innate preference for CMV-infected tomato plants. Bumblebees can be trained by differential conditioning (using solutions of 30% sucrose as a 'reward' versus 0.12% quinine as 'punishment') to distinguish between volatiles produced by different plants. Using the example of CMV-infected versus mock-inoculated tomato (*S. lycopersicum*, wild-type, labeled 'Sl WT'), bees initially have a 50% chance of making the 'correct' choice (sucrose: placed over mock-inoculated plants in A or over CMV-infected plants in B). Increasing success with each choice made is indicated by a rising learning curve, with overall ability to distinguish between plant-emitted volatiles analyzed after 100 choices. Where bees can perceive a difference in the volatiles emitted by two plants, they learn to identify sucrose rewards based on the association with plant volatiles, even as in Panel A when this opposes their innate preference for odor cues of CMV-infected plants (see Fig 1B). Data are shown pooled over all bees ($n = 8$ or $n = 9$) into successive groups of 10 choices, with error bars showing 95% binomial confidence intervals for the proportion of correct choices. In these experiments infected plants were inoculated with CMV strain Fny. The white curve shows the fitted binomial logistic model, with blue shading showing 95% confidence intervals on the fitted response. The χ^2 statistic and p-value for the likelihood ratio test assessing whether or not bees are able to learn are given at the bottom left of each panel.

doi:10.1371/journal.ppat.1005790.g002

One of the host molecules that interact with the 2b VSR is the Argonaute 1 (AGO1) 'slicer' protein. AGO1 is required for silencing directed both by short-interfering RNAs (which can be generated *de novo*) and by miRNAs, which are generated by a specific host endoribonuclease (DCL1) from miRNA precursor transcripts encoded by nuclear genes [23,24]. In differential conditioning assays, bumblebees were able to learn to distinguish between volatiles emitted by wild-type plants versus those emitted by *ago1* mutant plants (Fig 3H) and those emitted by *dcl1* mutant Arabidopsis plants (Fig 3I). However, bumblebees showed little or no ability to learn to distinguish between volatile blends emitted by *ago1* and *dcl1* mutant plants, indicating that the volatile blends emitted by plants of these two mutant lines were very similar (Fig 3J). Thus, the miRNA-directed silencing pathway regulates the emission of bee-perceivable volatile compounds. Double mutant *dcl2/4* plants are unable to generate CMV-derived short-interfering RNAs but are not affected in miRNA biogenesis. In CMV-infected *dcl2/4* plants a higher proportion of the 2b protein is available to bind AGO1 and inhibit its miRNA-directed activity [19], which is likely to enhance virus-induced changes in emission of bee-perceivable volatiles. In line with this, bumblebees were able to learn to distinguish between volatiles emitted by CMV-infected wild-type and *dcl2/4* double mutant Arabidopsis plants (Fig 3K). As an additional control we showed that bumblebees could not learn to distinguish between volatiles emitted by mock-inoculated plants covered by towers offering sucrose rewards or quinine punishments (Fig 3L).

CMV Infection Induces Quantitative and Qualitative Changes in the Volatile Blend Emitted by Tomato Plants

The responses of bumblebees to CMV-infected tomato plants that were hidden from the insects indicated that changes in the emission of volatiles were affecting bee behavior and were responsible for the innate preference of these insects for CMV-infected plants (Fig 1B). To confirm that CMV infection caused changes in the emission of VOCs, tomato plant headspace volatiles were collected and analysed by gas chromatography coupled to mass spectrometry (GC-MS). VOCs were collected from non-flowering mock-inoculated plants, plants infected with CMV-Fny and plants infected with the *2b* gene deletion mutant of CMV-Fny, CMVΔ2b. The emitted VOCs were distinct from each other when compared by principal component (PC) analysis on the relative intensity of ions (over 75 Da in size) within the samples (Fig 4A). PC1 explained 80.3% of the variation and discriminated between samples from mock-inoculated and CMV-infected plants, whereas PC2 discriminated between samples from mock-inoculated and CMVΔ2b-infected plants (Fig 4A). Thus, the VOC blend emitted by CMV-infected tomato plants was more distinct from that released by mock-inoculated plants than it was from the volatiles emitted by CMVΔ2b-infected plants. Nevertheless, VOC emission by

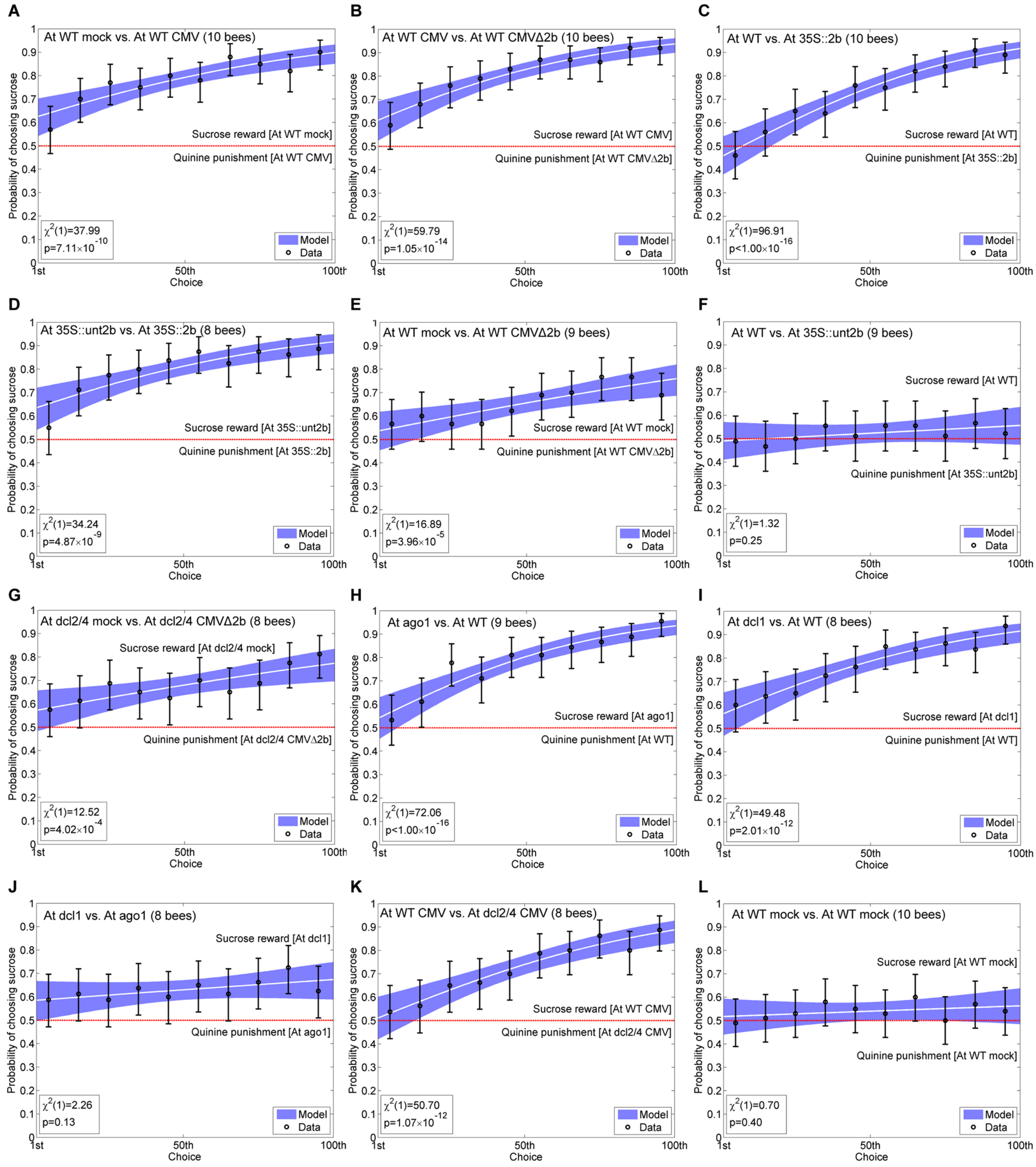

Fig 3. Bumblebees can perceive differences in volatiles emitted by Arabidopsis plants caused by CMV infection and by mutations affecting the microRNA pathway. Bumblebees can be trained by differential conditioning (learning curve experiments using solutions of 30% sucrose as a 'reward' versus 0.12% quinine as 'punishment') to respond to differences in volatiles produced by Arabidopsis plants (Panels A—L). Increasing success with each choice made is indicated by a rising learning curve, with overall ability to distinguish between plant-emitted volatiles analyzed after 100 choices per bee using between 8 and 10 bees as indicated in each panel (A—L). Using differential conditioning it was determined whether bumblebees could distinguish between volatiles emitted by wild-type (WT) *Arabidopsis thaliana* (At) plants after mock-inoculation (mock) or infection with CMV (strain Fny) (A) or CMV and the CMVΔ2b deletion mutant (B). Bees could readily learn to distinguish

between volatiles emitted by transgenic plants expressing the CMV 2b protein gene under control of the cauliflower mosaic virus 35S promoter (At 35S::2b) and volatiles emitted either by non-transgenic, WT plants (C) or by control-transgenic plants expressing an untranslatable *2b* gene construct (35S::unt2b) (D). Bumblebees did not learn efficiently to distinguish between volatiles emitted by mock-inoculated versus CMVΔ2b-infected wild-type plants (E), WT versus control-transgenic (35S::unt2b) plants (F), or *dcl2/4* double transgenic plants that had been mock-inoculated or infected with CMVΔ2b (G). Bees rapidly learned to distinguish between volatiles emitted by WT plants and plants harbouring mutant alleles for the *AGO1* (*ago1-25*) (H) or *DCL1* (*dcl1-9*) genes (I). (J) Bees showed little or no ability to learn to distinguish between volatiles emitted by *ago1-25* versus *dcl1-9* mutant plants. (K) Bumblebees readily learned to distinguished between *dcl2/4* double transgenic plants and WT plants infected with CMV. (L) Bees could not learn to distinguish between mock-inoculated WT plants. Data are shown pooled over all bees (n = 8 to 10) into successive groups of 10 choices, with error bars showing 95% binomial confidence intervals for the proportion of correct choices. The white curve shows the fitted binomial logistic model, with blue shading showing 95% confidence intervals on the fitted response. The χ^2 statistic and p-value for the likelihood ratio test assessing whether or not bees are able to learn are given at the bottom left of each panel.

doi:10.1371/journal.ppat.1005790.g003

CMVΔ2b-infected tomato plants was distinct from either mock-inoculated plants or CMV-infected plant VOC emission (Fig 4A), despite this mutant virus accumulating to markedly lower levels than CMV (S1 Fig).

Although CMV-infected plants were smaller than either mock-inoculated or CMVΔ2b-infected plants, the emission of the combined volatiles on a whole plant basis was similar between mock-inoculated and CMV-infected plants (Fig 4B). Indeed, expressing the emission of the combined VOCs by mass of tissue revealed that CMV-infected plants released greater quantities of volatiles compared to mock-inoculated and CMVΔ2b-infected plants (Fig 4C). Thus, despite being stunted, CMV-infected plants generated a greater total quantity of VOC than either mock-inoculated or CMVΔ2b-infected tomato plants.

Identification by GC-MS of the most abundant VOC by g dry weight of tomato plant tissue showed that terpenoids dominated the profile, with α-pinene, 2-carene, *p*-cymene, β-phellandrene and the sesquiterpene (*E*)-caryophyllene being apparent (Fig 4D and 4E). CMV infection caused quantitative changes in the profile of these VOCs; α-pinene and *p*-cymene emission increased markedly, whereas 2-carene and β-phellandrene did not, and (*E*)-caryophyllene almost disappeared from the profile (Fig 4E). Isomeric composition was not further determined than that stated here. When VOC emission was compared on a whole plant basis, α-pinene and *p*-cymene emission rates from CMV-infected plants appeared similar to mock-inoculated or CMVΔ2b-infected plants, while 2-carene and β-phellandrene levels appeared to be lower (although this was not statistically significant in a one-way ANOVA: Fig 4D). Bumblebees of a closely related species (*B. impatiens*) are known to be repelled by β-phellandrene and 2-carene [25]. Thus, lower emission values of these VOCs from CMV-infected plants may explain why bumblebees displayed an innate preference for CMV-infected tomato plants over mock-inoculated plants in free choice assays (Fig 1B). The VOC profiles of mock-inoculated and CMVΔ2b-infected plants were similar, although not identical (Fig 4A), and this could explain the bees' lack of preference in free choice assays (Fig 1B).

CMV Infection Inhibits Seed Production but Accelerates Flowering in Tomato

Domesticated tomato plants are often said to be self-fertilizing. However, optimal self-fertilization requires sonication of the flower to release pollen from the anthers onto the stigma, which is provided either by buzz-pollination from a bee (typically a bumblebee) or simulated buzz-pollination using mechanical vibration [5]. This is illustrated in Fig 5A, which shows how mechanical buzz-pollination of flowers increased seed production by around a third. Seed production by tomato was very dramatically decreased in plants infected with CMV-Fny to less than 10% of the yield in mock-inoculated plants (Fig 5A). Remarkably, artificial buzz-pollination of flowers of CMV-infected plants rescued seed production to a significant degree with seed numbers reaching approximately half the level seen for non-buzzed flowers of mock-

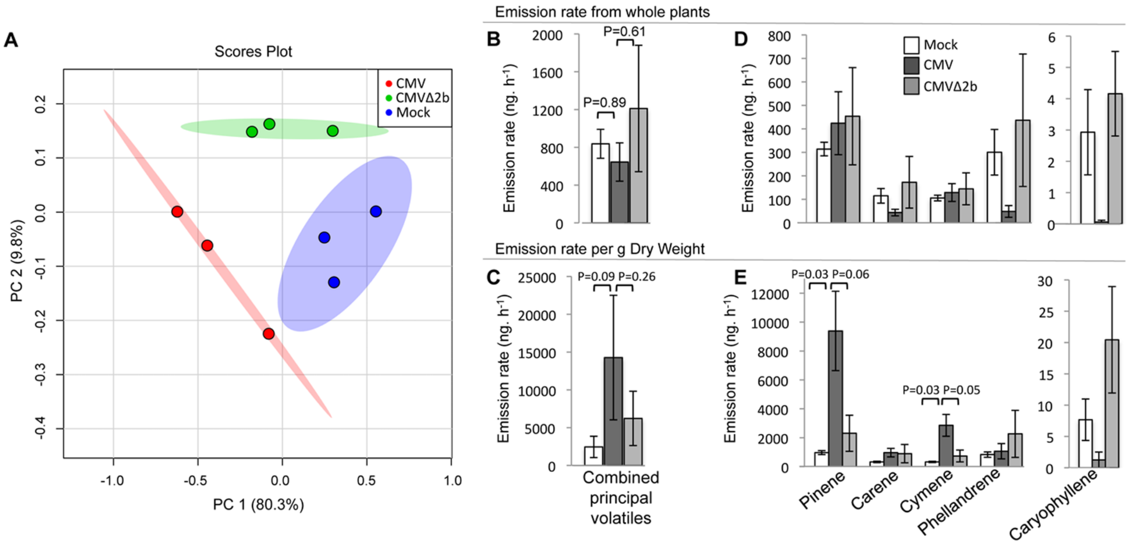

Fig 4. Virus infection induced quantitative and qualitative changes in the emission of volatile organic compounds by tomato plants. (A) Score scatter plot from principal component (PC) analysis of m/z values (binned to 1.0 Da) obtained by gas chromatography-mass spectrometry of samples of volatile organic compounds (VOCs) collected by dynamic headspace trapping from tomato plants that had been mock-inoculated (blue), infected with CMV (strain Fny) (red) or CMVΔ2b-infected (green). The analysis shows discrimination between all three treatments. The percentage of variation of the data explained by PC1 and PC2 is in parentheses (80.3 and 9.8%, respectively). (B) Whole plant total emission rate ($ng.h^{-1}$) of the combined (most abundant) volatiles is similar for mock-inoculated and virus-infected plants. (C) VOC emission rate ($ng.h^{-1}$) per gram dry weight of the combined (most abundant) volatiles is highest from CMV-infected plants compared to mock-inoculated and CMVΔ2b-infected plants. (D) Whole plant VOC emission rate ($ng.h^{-1}$) for the five most abundant volatiles from mock-inoculated, CMV-infected and CMVΔ2b-infected tomato plants. (E) VOC emission rate ($ng.h^{-1}$) per gram dry weight of the five most abundant volatiles from mock-inoculated, CMV-infected and CMVΔ2b-infected tomato plants show that pinene and cymene emission are significantly higher in CMV-infected plants compared to mock-inoculated and CMVΔ2b-infected plants. The mean VOC emission values for combined or individual volatiles are presented ($n = 3$ plants per treatment). Error bars represent standard error of the mean. The level of significance is shown by a p-value calculated with one-way ANOVA and post hoc Tukey HSD testing.

doi:10.1371/journal.ppat.1005790.g004

inoculated plants and about 6- to 7-fold greater than the number of seeds produced in non-buzzed, CMV-infected plants. The difference in seed yield between mock-inoculated and CMV-infected plants that had been vibrated was less marked than between non-buzzed, mock-inoculated and CMV-infected plants (Fig 5A).

Although CMV-infected plants produced fewer seeds, the mass of individual seeds was unaffected by infection and was not affected whether or not flowers were vibrated (Fig 5B). Additionally, the number of flowers produced by CMV-infected plants was similar to the number produced by mock-inoculated plants, and tomato flower morphology was also not markedly altered by infection (S2 Fig). Overall plant growth was stunted by CMV infection (S2 Fig) but, interestingly, virus infection appeared to accelerate the appearance of flowers by a small but statistically significant degree (S2 Fig). A recent report indicated that flowers of squash (*Cucurbita pepo*) plants infected with the potyvirus zucchini yellow mosaic virus yielded decreased quantities of pollen [26]. However, we found no significant differences in the quantity or viability of pollen released from mock-inoculated and CMV-infected tomato flowers (S3 Fig).

The Effects of CMV on Bumblebee-Mediated Pollination of Tomato Plants

We investigated the effects of CMV infection on bumblebee-mediated pollination under glasshouse conditions in which the insects could see and interact with flowers (Fig 6). A European

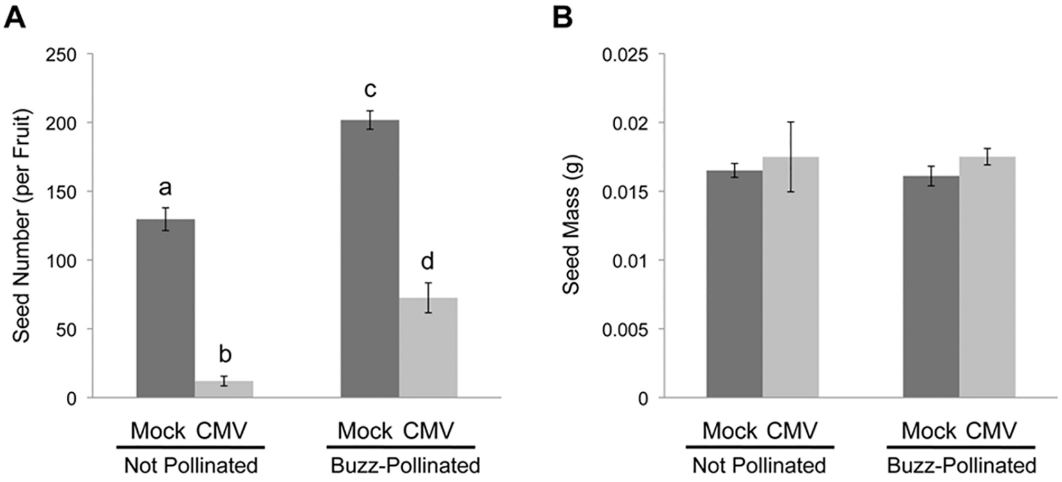

Fig 5. Impacts of CMV infection and artificial buzz-pollination on tomato seed production. (A, B) Tomato plants infected with cucumber mosaic virus (CMV) (strain Fny) produced fewer seeds (A) than mock-inoculated (Mock) plants but seed mass was not affected (B). However, buzz-pollination significantly enhanced seed production (A) but not seed mass (B). Artificial buzz pollination was achieved by touching flower stalks of matured flowers with an electrical toothbrush. This was done three times just before, during and after apparent flower maturation to ensure efficient buzz-pollination. Successful buzz-pollination was noted by observing pollen release from the anther cone. Letters indicate significant differences. A) Mean seed number per fruit (two-way ANOVA: infection status, $F(1,10) = 220.9938$, $p = 3.811e-08$; pollination treatment, $F(1,10) = 61.5886$, $p = 1.393e-05$; infection status x pollination treatment, $F(1,10) = 0.4701$, $p = 0.5085$). B) Mean mass per seed (two-way ANOVA: infection status, $F(1,8) = 0.9291$, $p = 0.3633$; pollination treatment, $F(1,8) = 0.0030$, $p = 0.9577$; infection status x pollination treatment, $F(1,8) = 0.0825$, $p = 0.7812$). Error bars represent the standard error of the mean; $n = 3$ plants per experiment.

doi:10.1371/journal.ppat.1005790.g005

CMV isolate, PV0187, which is 99% identical in RNA sequence to CMV-Fny and which encodes a 2b VSR that is identical in amino acid sequence to that of CMV-Fny (S4 Fig), was used for these experiments in order to comply with UK quarantine and containment regulations. CMV-PV0187 had similar effects on growth of tomato plants as CMV-Fny (S5 Fig) and volatiles emitted by tomato plants infected with CMV-PV0187 were attractive to bumblebees in the free choice assay (Fig 1B).

When CMV-infected and mock-inoculated tomato plants were exposed to bumblebees, a higher proportion of the insects made their initial floral visits to CMV-infected plants and spent longer sonicating the flowers of CMV-infected plants (S6 Fig). As had been seen for artificial buzz-pollination (Fig 5), when bumblebees buzzed flowers, seed yield was increased (Fig 6). For CMV-infected plants, when bees did not visit flowers or where flowers were on plants not exposed to bees (untouched plants), the seed yield was significantly decreased (Fig 6). However, although CMV infection decreased seed number in fruits derived from unvisited flowers, buzz-pollination by bumblebees negated this effect; indeed, bee-pollinated flowers on CMV-infected plants yielded fruit that contained seed numbers similar to those found in fruit that developed from bee-pollinated flowers on mock-inoculated plants (Fig 6). The results imply that there was greater buzzing activity on flowers of CMV-infected plants (S6 Fig), resulting in a greater amount of seed production.

Mathematical Modeling: Pollinator Preference for Infected Plants Could Impede Evolution of Resistance

We have seen that under controlled conditions CMV infection made tomato plants more attractive to bumblebees (Fig 1B). We also found that although infected plants yielded fewer seeds, simulated buzz-pollination could to some extent rescue seed production (Fig 5A) and

when bees were allowed access to CMV-infected plants they caused a greater increase in seed production per fruit compared to simulated buzz-pollination (Fig 6). The results obtained with this domesticated plant under controlled conditions prompted us to wonder what would be the consequences for a wild buzz-pollinated plant growing under natural conditions, if virus infection resulted in greater pollinator visitation and/or seed production and whether this might result in any benefits for the host plant or the virus.

To investigate this idea further we developed a mathematical model to test whether increased pollinator service to virus-infected plants could allow genes for virus susceptibility to persist in a host plant population, despite the significant fitness cost of infection for plants as female (seed producing) parents (*cf*. Figs 5 and 6). Our model tracks the long-term dynamics of the interaction between resistant and susceptible phenotypes in a population of annual plants (see also Materials and Methods). We focused on resistance as a dominantly inherited

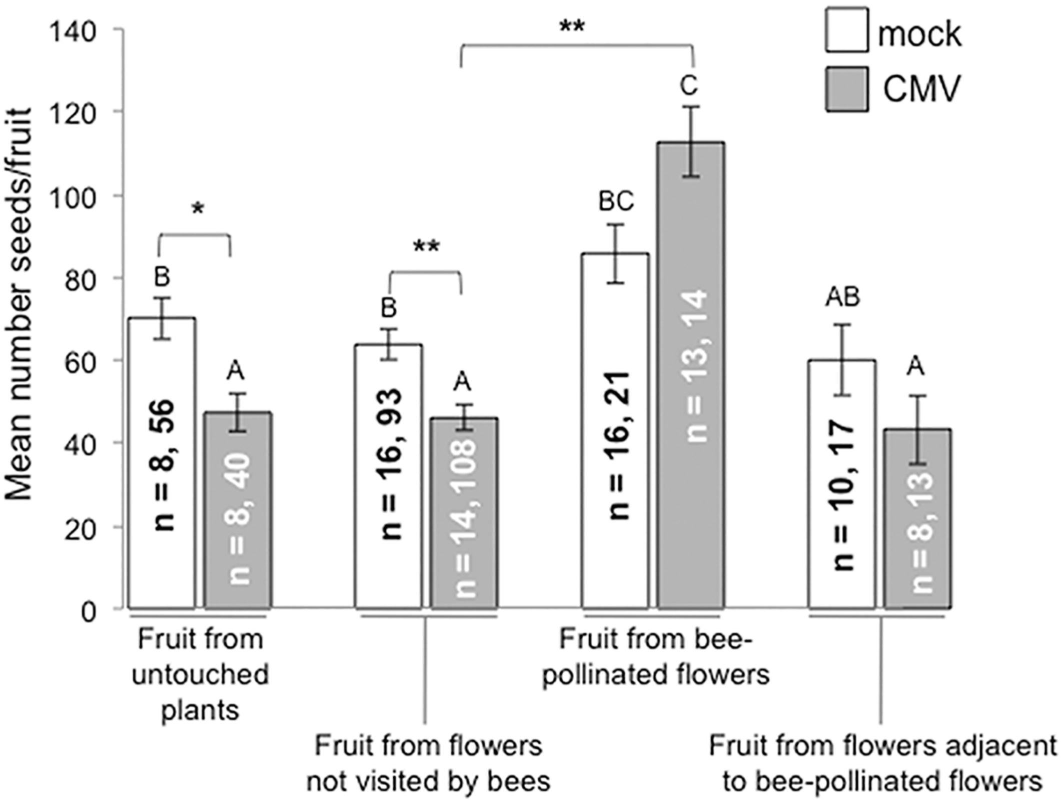

Fig 6. Bumblebee-mediated pollination of CMV-infected and mock-inoculated tomato plants. Within a flight arena under glasshouse conditions bumblebees were allowed to forage directly on flowering plants that had been mock-inoculated or infected with CMV (strain PV0187) and fruits were allowed to develop on these plants and later harvested. Fruits were categorized according to whether they were derived from flowers that had not been buzz-pollinated by a bumblebee (fruit from flowers not visited by bee) or from flowers that had been buzz-pollinated (fruit from bee-pollinated flowers). A further category of fruit was from flowers that had not been buzz-pollinated, but had been adjacent to buzz-pollinated flowers (fruit from flowers adjacent to bee-pollinated flowers). Fruits were also harvested from 8 mock-inoculated and 8 CMV-infected plants that had been placed in the flight arena and had otherwise experienced the same growth conditions but were never exposed to bees (fruit from untouched plants). Numbers of seeds per fruit differed between treatments (one-way ANOVA, $F(7,459) = 12.34$, $p<10^{-13}$). In control plants not exposed to bumblebees, CMV significantly lowered the number of seeds per fruit by over 30% ($p = 0.013$ post-hoc Tukey test). Natural buzz-pollination by bumblebees raised the seed number in fruit from both mock-inoculated and CMV-infected plants and remarkably had a more beneficial effect on CMV-infected plants in that the seed yield per fruit matched that of the mock-inoculated plants. Different letters (A, B, or C) are assigned to significantly different results (post-hoc Tukey tests, $p< 0.05$, *, $p< 0.01$, **). Histogram bar labeling: n = number of plants, number of fruits. Error bars are standard error around the mean seed number.

doi:10.1371/journal.ppat.1005790.g006

trait and attached no fitness penalty to the presence of resistance, which is a conservative approach given that recessive resistance is a commonly observed antiviral defense mechanism and that resistance may incur fitness costs in the absence of infection [27]. We assume infected susceptible plants produce fewer seeds, with the parameter δ controlling the proportionate number of viable seeds produced per fertilized ovary on a virus-infected plant. However, we also assume that, all other things being equal, an individual visit by a pollinator is ν times more likely to be to a flower on an infected *versus* an uninfected plant. This pollinator bias makes infected plants more likely to reproduce as both male (pollen donor) and female (seed producing) parents, potentially out-weighing the deleterious effect of infection on seed production.

We focus initially on the trade-off between pollinator bias (ν) and reduction in seed production (δ), for different levels of pollinator service (which we parameterize via γ, the mean number of pollinator visits per flower over the plant's reproductive season). In indicative examples of both low ($\gamma = 0.25$) and high ($\gamma = 2.5$) pollination regimes, (ν, δ) parameter space can be divided into three regions: resistance takes over in the long-term, susceptibility takes over in the long-term, or resistant and susceptible plants coexist (Fig 7A and 7B). For both values of γ, at high values of ν and δ (i.e. if infected plants are strongly preferred by pollinators but do not suffer a great reduction in seed production), then genes conferring susceptibility will take over in the plant population. For low values of ν and δ the situation is reversed, and resistance is favored. At intermediate values of ν and δ, resistant and susceptible plants coexist.

For fixed baseline values of $\nu = 3.0$ and $\delta = 0.5$, the proportion of susceptible alleles in the population first increases then decreases as the level of pollinator service (γ) is increased (Fig 7C). At very low values of γ, although virus-infected plants benefit from additional pollinator service on both male and female sides, the vast majority of fertilizations do not involve pollinator visits (instead being via self-pollination). The cost to susceptible plants of reduced seed production as female parents is therefore more important than increased pollinator visitation, and so virus resistance takes over. As γ is increased, the proportion of fertilizations caused by pollinators goes up, which allows the benefits to virus-infected plants on both male and female sides to outweigh the cost of infection, and so the genes for susceptibility are favored. As γ is increased still further, the benefit on the female side becomes smaller (since pollinator visitation is not limiting and almost all ovules are fertilized), but on the male side proportionately more pollen still comes from infected plants. For these values of the parameters, alleles conferring virus susceptibility persist in the plant population, but at reduced density. The maximum density of susceptible genotype plants is therefore realised at intermediate pollinator densities.

The broad pattern of a rise then fall in the proportion of plants carrying the susceptible allele is repeated for a range of values of the proportion of susceptible plants that are infected (i.e. the parameter α in our model: Fig 7D). However, for our default parameterization at low levels of infection the eventual fall with increasing pollinator levels is not apparent, and susceptible plants exclude resistant plants even for very high values of γ.

A full sensitivity scan around default parameter values $\nu = 3.0$, $\delta = 0.5$, $\gamma = 1.0$, $\sigma = 0.5$, $\varphi = 0.75$ and $\alpha = 0.5$, (Fig 8; S7 Fig) shows the behaviour of the model over large regions of parameter space. The susceptible genotype is able to persist under many combinations of parameters. Our model therefore suggests preferential visitation of infected plants by pollinators could in principle provide a robust mechanism allowing susceptible genotype plants to be retained in the host population for a wide range of conditions.

Discussion

Infection with CMV altered the volatile profile of tomato plants and made them more attractive to bumblebees, indicating that these insects possess an innate preference for the blend of

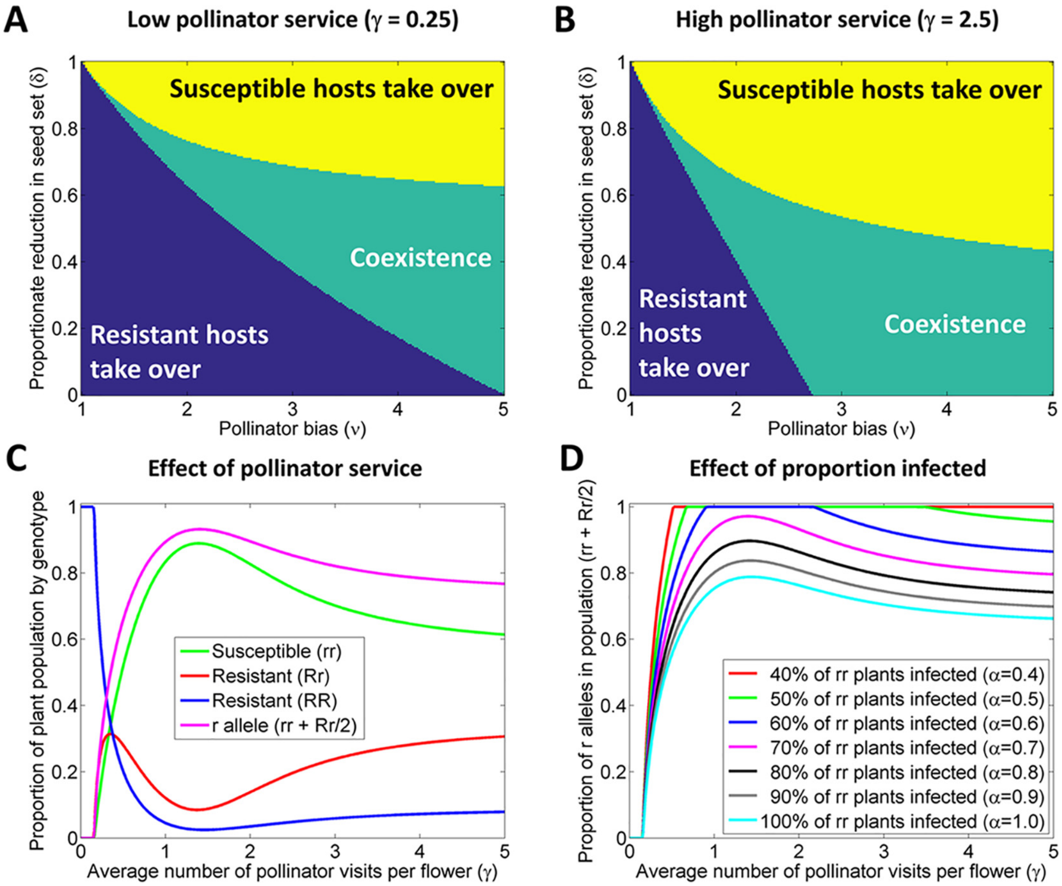

Fig 7. Persistence of genes for virus susceptibility depends on the balance between positive and negative effects of infection on reproduction. (A) and (B). Whether or not susceptible genotype plants take over (yellow), coexist with resistant plants (green) or are eliminated in favor of resistance (blue), depends on the balance of the pollinator bias to infected plants (ν), the reduction in seed set by infected plants (δ), and the mean number of pollinator visits per flower (γ). Other parameters are fixed at default values: proportion of flowers that self-fertilize without being visited by a pollinator (σ = 0.25), probability that flowers that are visited by a pollinator are cross-pollinated (φ = 0.75) and the proportion of virus-susceptible plants that are infected (α = 0.75). (C) For fixed pollinator bias (ν = 3) and reduction in fertility of infected plants (δ = 0.5), the long-term genotypic structure of the plant population depends on the number of visits by pollinators (γ). (D) The exact form of the response to γ depends on the proportion of susceptible plants that become virus infected (α).

doi:10.1371/journal.ppat.1005790.g007

volatile compounds emitted by CMV-infected tomato. Although bumblebees showed no innate preference for CMV-infected or mock-inoculated Arabidopsis plants, differential conditioning experiments showed that bumblebees were able to perceive alterations in volatiles emitted by these plants. Experiments with the *2b* gene deletion mutant virus, CMVΔ2b, in tomato and Arabidopsis, and with *2b*-transgenic and *ago1* and *dcl1* mutant Arabidopsis plants, implicate small RNA pathways in the regulation of the production of bee-perceivable volatile compounds.

The inability of bees to learn to effectively distinguish between volatiles emitted by *ago1* and *dcl1* mutant plants causes us to conclude that miRNAs are the predominant class of small RNAs involved in regulating the metabolism of bee-perceivable compounds. The rationale for this conclusion is that AGO1, a target for the CMV 2b VSR, utilizes both short-interfering RNAs and miRNAs to guide RNA cleavage, while DCL1 is involved in miRNA biogenesis but is not involved in production of short-interfering RNAs (see refs. [23, 24] and references

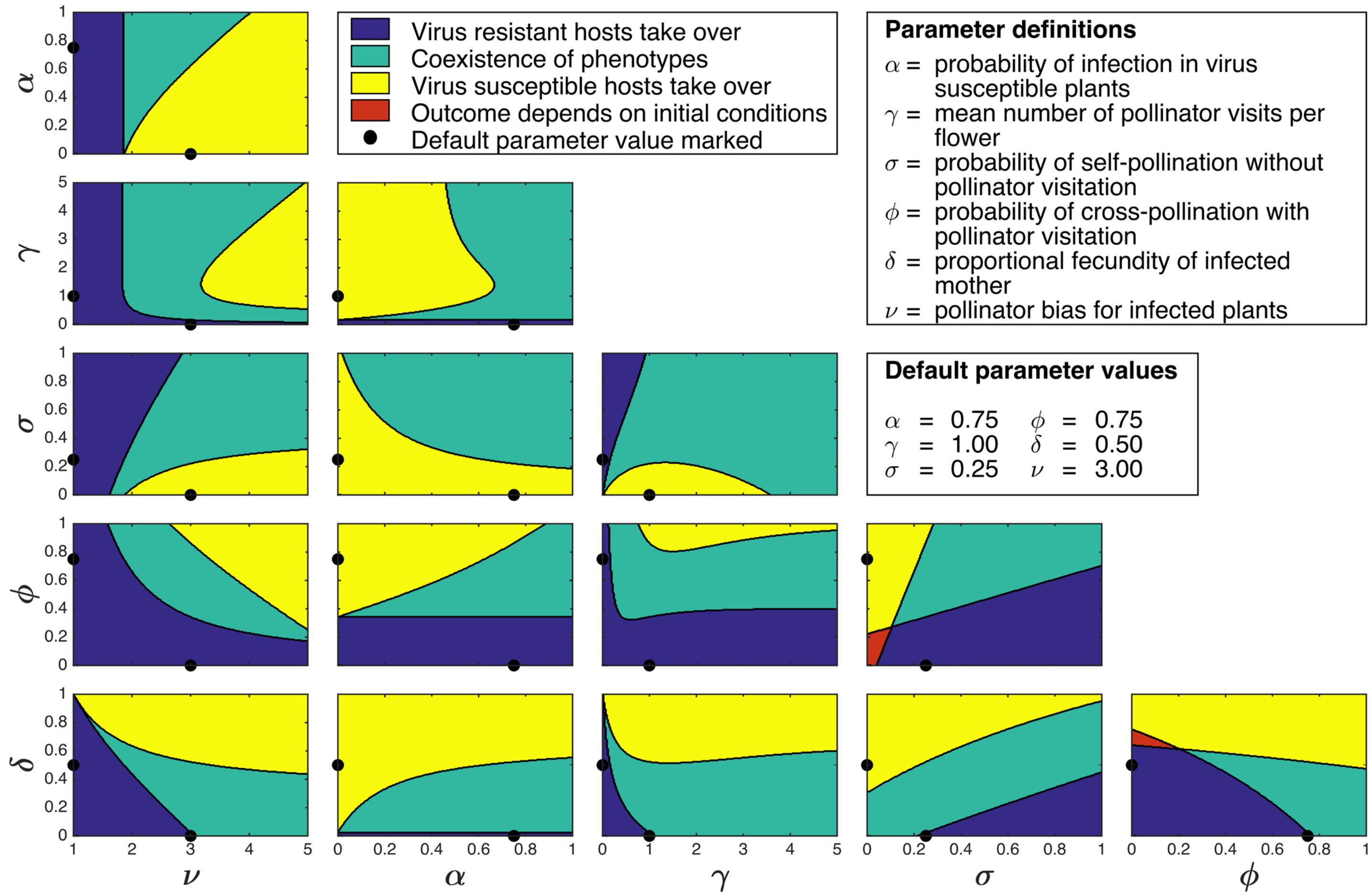

Fig 8. Virus susceptible plants persist across a broad range of parameter values. A full two-way sensitivity analysis of the model, showing the effect of independently changing pairs of parameters (all other parameters fixed). The virus susceptible genotype takes over (yellow) or co-exists with the resistant genotype (green) across a large proportion of parameter space. All pair-wise combinations of two parameters are shown: dots on each axis show default values of each parameter.

doi:10.1371/journal.ppat.1005790.g008

therein). As far as we are aware, an effect of miRNAs on plant volatile production (presumably through regulation of stability or translation of specific plant mRNAs) has not been previously reported. The work also indicates that olfactory signals emitted by non-floral tissue may have a more important effect than previously thought in plant-bee interactions and may play roles in bee attraction, presumably at longer ranges than visual features such as the optical or tactile qualities of flowers. Thus, foliar volatile signals may affect bee choices or synergize with and reinforce visual floral cues, as has been seen with floral odors [28, 29].

How do changes in the output of volatiles increase the attractiveness of CMV-infected plants for bumblebees? Much of the existing bee perception literature is focused on the effects of visual stimuli (e.g. color and other optical properties of flowers [14]), whereas the effects of olfactory stimuli have been relatively neglected. But it is known, for example, that the VOC output from flowers decreases after they have been pollinated [12]. Pollination can also trigger qualitative changes in the volatile blend. For instance, following pollination by bees, blueberry (*Vaccinium corymbosum*) flowers emit an increased proportion of their volatiles as (*E*)-caryophyllene [30]. It is thought that decreased volatile emission by pollinated flowers decreases their saliency to bees and prevents damage from over-visitation [12] and a similar explanation

was offered by Rodriguez-Saona and colleagues [30] to explain the post-visit increase in (*E*)-caryophyllene emission. In the case of tomato plants infected with CMV, it may be that the virus is both 'turning up the volume' of plant volatile emission (making these plants more apparent to the bumblebees) whilst 'tuning' volatile blend composition so as to diminish levels of a signal ((*E*)-caryophyllene), that at higher levels might indicate a previous bee visitation, and levels of β-phellandrene and 2-carene that might discourage visitation [25].

When the bumblebees were allowed access to flowering tomato plants under glasshouse conditions we found that buzz-pollination by bumblebees was more effective at enhancing seed yield on CMV-infected plants. This result suggests that additional foliar or floral cues, for example visual or tactile stimuli, do not negate the effects on the bees of CMV-induced changes in volatile emission.

It is possible that our findings may have implications for transmission of viruses vectored by bees. However, pollinators transmit very few viruses and CMV is not one of them (discussed on page 522 in reference [31]). Nevertheless, is it possible that a virus that is not bee transmitted gains some advantage by re-paying a susceptible host by altering its volatile cues to attract pollinators? In our mathematical model it was assumed that a hypothetical population of wild plants included some hosts that possessed genetic resistance to the virus. It might then be assumed that pathogen-imposed selection pressure would favor the takeover of the plant population by any plants possessing one or more resistance genes. This outcome, causing a decrease in the population, or even the extinction, of susceptible plants would clearly not be beneficial either for the pathogen or for the susceptible hosts. However, our mathematical model shows that in the case where pollinators show increased bias towards pathogen-infected plants, the increased reproductive success of infected plants means that the outcome might be different. Thus, it is plausible that if the attractiveness of infected plants to pollinators is increased, this might inhibit or negate the selective advantage of resistant individuals and prevent them from taking over the population (represented conceptually in Fig 9). We also noted that CMV infection accelerated the appearance of flowers in tomato. If such an effect occurred in a wild plant population, it is conceivable that this may give infected, susceptible plants a further advantage over resistant or uninfected plants in the competition for limited pollinator services. Indeed, there are examples in which earlier flowering increases pollination and enhances yield (for example in the oil crop plant *Echium plantagineum*)[32]. However, the relationship between flowering time and pollination is complex and there may be environments in which it is more advantageous for plants to flower in a concerted fashion. However, in certain contexts earlier flowering may result in flowers being produced before pollinators are available (reviewed in [33]).

At this stage, it may be imprudent and premature to propose that increased pollinator attraction to infected, susceptible hosts represents some sort of specific viral strategy to inhibit selection for resistance, and there are difficulties in envisaging how this might initially arise. However, it seems plausible to suggest that in principle increased pollinator attraction to virus-infected plants could favor the persistence of susceptible plants in the environment and this could be seen as payback or compensation to the host. It is worth noting that other forms of payback by viruses to their hosts have been observed in a number of systems. This has led to the suggestion that our general view of viruses has been overly colored by their pathogenic properties and that we should view them as symbionts in the classical sense (*viz*. on a spectrum that ranges from parasitic to mutualistic [34]). For plant viruses it has been shown that virus infection can enhance the endurance of susceptible host plants to drought or in one case to cold [35, 36] and that plants of several species were protected from herbivory by virus infection [37–40]. It has been suggested that resistance to drought is a conditional phenotype that could act as a payback to the host. In the case of CMV-induced drought resistance in Arabidopsis

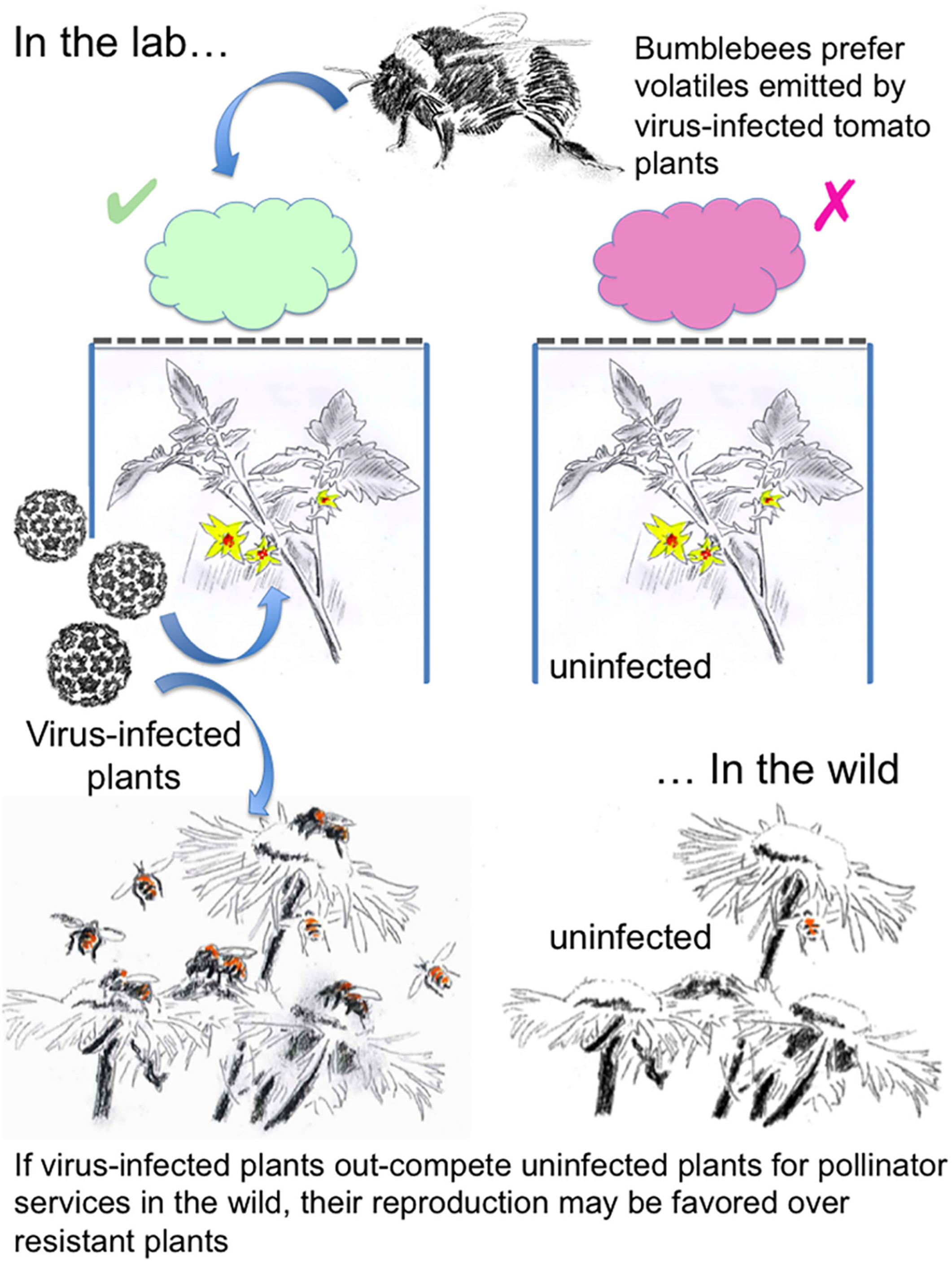

Fig 9. Hypothesis: Pollinator preference for virus-infected plants could provide a payback to virus-susceptible hosts. Under experimental conditions, bumblebees showed an innate preference for volatiles emitted by tomato plants infected with CMV (upper section of cartoon). We speculate that if similar phenomena occur under natural conditions in wild plant populations, this may pay back susceptible host plants by encouraging pollinator visitation. Mathematical modeling suggests that under some conditions this may result in increased production of virus-susceptible offspring and if pollinator preference for infected susceptible plants was sufficiently strong, this could outweigh underlying strong selection pressures favoring the emergence of virus resistance.

doi:10.1371/journal.ppat.1005790.g009

and other plants [35, 36] and in the present study, in which CMV enhances a tomato plant's attractiveness to bumblebees, we may be seeing examples of 'extended phenotypes'. An extended phenotype emerges from the action of a parasite gene when it alters the phenotype of a host; potentially to the benefit of the parasite [41]. In both examples, drought resistance in Arabidopsis [36] and pollinator attraction in tomato (the present study), the parasite gene controlling these extended phenotypes is the CMV *2b* gene. A potential result of these extended phenotypes would be to increase the odds of continued survival of susceptible host plant populations, which would be beneficial to both host and pathogen.

Our mathematical modeling results indicated that, for the areas of the parameter space that are most salient to our experimental findings, the most likely outcome of long-term selection would be coexistence of resistant and susceptible genotypes, i.e. the long-term maintenance of *R* gene polymorphisms. Several mechanisms have been proposed that could contribute to the maintenance of balanced *R* gene polymorphisms such as the ratio of costs *versus* benefits of resistance, and diffuse interactions between hosts and attackers [27,42,43]. Our data suggest that the enhanced attraction of pollinators to infected susceptible plants might add to these mechanisms and contribute to the long-term maintenance of *R* gene polymorphisms in insect-pollinated species.

Production of many important crops depends on bee-facilitated pollination. Worryingly, bee populations are endangered by disease, environmental change [44,45] and, more controversially, by anthropogenic factors [46]. To mitigate the ensuing loss of pollination activity requires among other things a deeper understanding of the mechanisms shaping bee-plant interactions. Our data show that non-floral plant volatiles can be perceived by bumblebees and affect their behaviour and that emission by plants of bee-perceivable compounds is regulated in part by miRNA activity. This information may be useful in developing strategies to increase pollinator services for crops under conditions of cultivation, as well as for a better understanding of the interplay of plant pathogens, wild plants and pollinators under natural conditions.

Materials and Methods

Viruses and Plants

Plants used were *Arabidopsis thaliana* (Heynh.) accession Col-0 and *Solanum lycopersicum* (L.) cv. Moneymaker (Suttons Seeds Ltd., Paignton, UK). Plants were grown in a growth chamber at 22°C in M3 compost (Levingtons Ltd., Northampton, UK). Tomato and Arabidopsis plants were grown under 16hr light/8hr dark and 8hr light/16hr dark photoperiods, respectively. Fny-CMV [47], Fny-CMVΔ2b [48], the *2b*-transgenic plant line 2.30F [49], and the *dcl1-9*, *dcl2/4*, and *ago1-25* mutant plant lines have been described elsewhere [19,50,51]. CMV isolate PV0187 was obtained from the German Collection of Microorganisms and Cell Cultures (DSMZ, www.dsmz.de). RNAs1, 2 and 3 of CMV isolate PV0187 were sequenced and submitted to GenBank under accession numbers KP165580, KP165581, and KP165582, respectively. Inoculations were carried out at the seedling stage and were performed as described previously [49]. Plants were used in experiments when the virus had spread systemically and infection was confirmed routinely by double-antibody sandwich enzyme-linked immunosorbent assays (BioReba, Reinach, Switzerland). Quantification of CMV and CMVΔ2b RNA accumulation was carried out as previously described [52]. Leaf tissue from systemically infected leaves was harvested at 10 and 18 dpi. Total RNA for reverse transcription coupled polymerase chain reaction analysis was extracted using an RNeasy Plant Kit (Qiagen) and treated with TURBO-DNase (Ambion) according to the manufacturers' instructions. Reverse transcription was carried out with 0.5 μg total RNA using Goscript (Promega) with random hexamer

primers according to the manufacturer's instructions. Following the reaction, cDNA was diluted 1/10 for subsequent use. Semi-quantitative PCR was performed using Biomix Red (Bioline) and products were separated electrophoretically on a 1.5% agarose gel. Reverse transcription coupled to quantitative polymerase chain reaction analysis was performed using SYBR Green JumpStart Taq ReadyMix (Sigma) in 15 μl reactions according to the manufacturer's instructions. Reactions were performed in triplicate. Primers described in [52] were designed against the conserved 3' non-translated regions of the CMV genomic RNAs and the stable transcript *elongation factor 1 alpha* (*EF1α*) was used as the reference RNA. Data were analyzed using LinRegPCR to give Ct values. Relative viral RNA accumulation was calculated using ΔΔCt methodology, incorporating the *EF1α* transcript to control for variation in loading [53].

Bumblebees and Arena Design for Olfactory Studies

Bombus terrestris (L.) colonies (obtained from Syngenta-Bioline, Leicester, UK and Koppert Biological Systems, Berkel en Rodenrijs, The Netherlands) were connected by gated transparent tubing to flight arenas with the dimensions 72 x 104 x 30 cm [22] containing 11 cm tall feeding towers (to conceal plants) formed from black card sitting within 'Aracon' bases (Lehle, Roundrock, TX), roofed by plastic mesh supporting a microcentrifuge tube lid (Fig 1A) containing sucrose solution. Tower height was selected because bumblebees cannot effectively resolve visual cues beyond 10 cm [54]. Seven days prior to carrying out conditioning or free choice assays bees were allowed to feed on sucrose solution from cups placed on empty towers for three days to familiarize them with the arena. Foraging bees were marked on the thorax with water-soluble paint and used once.

Differential Conditioning and Free-Choice Preference Assays

Initially, cups on towers offered 30% sucrose, conditioning bees to associate towers with a reward. For differential conditioning and free-choice experiments, five plants per treatment group were individually covered by towers. For differential conditioning experiments, towers hiding plants from one treatment group provided 0.3 ml quinine hemisulfate (0.12%), whilst the others offered 0.3 ml of 30% sucrose. Individual foraging bumblebees were released into the arena and allowed to forage until satiated. Aborts following landing or hovering over towers offering quinine or drinking on towers offering sucrose were scored as correct choices. Between foraging bouts, towers were re-arranged randomly to inhibit spatial learning and meshes cleaned (30% ethanol) to remove scent marks. One hundred choices for each bee tested for each pair-wise comparison were recorded. In free-choice preference assays towers covering plants from both treatment groups offered equal sucrose rewards and only the first feeding choice was recorded.

The learning curve data were analysed using binomial logistic regression [55]. The experimental protocol did not record individual choices made by the bees, but instead the number of 'correct' choices made by each bee was grouped into sets of 10 successive choices for ease of scoring. Exploratory analyses suggested no pronounced differences between individual bees within treatment groups, and so we fitted the following fixed effect model to these data

$$b_{ij} \sim \mathrm{Bin}(10, p_i),$$
$$\log\left(\frac{p_i}{1-p_i}\right) = \alpha_0 + \alpha_1(i - 0.5),$$

where b_{ij} is the number of correct choices made by the jth bee in its ith set of ten choices, p_i is the probability of choosing correctly in each successive batch of ten choices, and where α_0 and α_1 are the parameters to be estimated. We used Hosmer-Lemeshow tests to assess model

goodness-of-fit [56]: in all cases there was no evidence for lack-of-fit. We therefore went on to assess whether the parameter α_1 was different to zero via a likelihood ratio test against the simpler nested model with α_1 fixed to be zero [57]. Since the parameter α_1 controls how the (logit) of the probability of making a correct choice p_i increases with i, positive values of α_1 correspond to the bees 'learning' over time.

Any systematic differences in the rate at which bees learn between pairs of experiments was assessed by simultaneously fitting a single regression model to the results of both experiments, allowing the probabilities of making a correct choice to depend on the experiment via

$$\log\left(\frac{p_i(E)}{1-p_i(E)}\right) = \alpha_0 + (\alpha_1 + \alpha_2 E)(i - 0.5),$$

in which E is an indicator variable which is equal to zero for the first experiment, and equal to one in the second experiment. A value $\alpha_2 \neq 0$ corresponds to bees learning at a different rate in the different experiments: again, this was tested via a likelihood ratio test against the simpler nested model in which α_2 was fixed to be zero.

Pollination Experiments

Artificial buzz-pollination was carried out using an electrically actuated toothbrush ('Oral-B': Proctor and Gamble, Cincinnati, USA). Mean seed mass was obtained by dividing the mass of seeds by the total seed number for a total of five fruits per plant, with three plants per treatment group. Pollen viability was assessed by staining with fluorescein diacetate [58] and pollen grains viewed under blue light and bright field using an epi-fluorescent microscope (DMRXA, Zeiss) connected to a digital camera (DFC425, Zeiss).

For bumblebee pollination experiments two-week-old tomato seedlings were inoculated with CMV (isolate PV0187) or mock-inoculated and grown in a controlled environment room for 4 weeks. At this time, the plants began flowering and were transferred to a glasshouse. Two weeks later single bumblebees (released from a small flight arena) were allowed to buzz pollinate flowers on three mock-inoculated and three CMV-infected tomato plants within a larger flight arena (125 x 370 x 90cm, H x W x D) constructed from nylon netting (S8 Fig) (JoTech-Insectopia Ltd., Austrey, UK). Two inflorescences of two to three flowers per plant were left accessible to the bee (any more inflorescences were covered with a paper bag). When each bee had made 10 visits to flowers (or had ceased pollinating), any buzz-pollinated flowers were labeled with a jeweler's tag and all plants that had been visited by the bee were removed from the arena and replaced with another. A new bee was then released from the small arena into the larger arena containing plants. In total, 8 bees freely pollinated flowers from 17 mock-inoculated and 14 CMV-infected tomato plants. Bumblebee visitation to mock-inoculated versus CMV-infected plants was noted and, using a stopwatch, the duration of flower sonication was recorded for each bee. The plants were left in the greenhouse for a further 8 weeks to allow fruits to develop. Further flower development on the plants was permitted.

To release seeds, fruits were harvested individually into 60 ml screw-cap pots and left to ferment for 1–2 weeks before washing and counting. Fruits were either from flowers that were not buzz-pollinated by a bumblebee (fruit from flowers not visited by bee) or from flowers that were buzz-pollinated (fruit from bee-pollinated flowers). A further category of fruit was from flowers that were not buzz-pollinated, but were adjacent to fruit from buzz-pollinated flowers (fruit from flowers adjacent to bee-pollinated flowers). Fruits were also harvested from eight mock-inoculated and eight CMV-infected plants that were not exposed to bees in the flight arena, but had otherwise experienced the same growth conditions as the plants used in the bee pollination experiment (fruit from untouched plants).

Volatile Analysis

Headspace volatiles were collected from tomato plants (4 weeks-old) by dynamic headspace trapping over a period of 24 hours onto Porapak Q filters [50 mg, 60/80 mesh size, Supelco (Sigma-Aldrich)] as described by Beale and colleagues [59]. The tomato plants were contained in a 1.0 liter bell jar clamped to two semi-circular metal plates with a hole in the center to accommodate the stem. Charcoal-filtered air was pumped in at the bottom of the container at a rate of 750 $ml.min^{-1}$ and drawn out through the Porapak Q filter at the top, at a rate of 700 $ml.min^{-1}$. Leaf fresh weight and dry weight were measured to enable normalization of the volatile abundance. Trapped organic chemicals were eluted from the Porapak Q filter with diethyl ether for analysis by gas chromatography coupled to mass spectrometry (GC-MS). For initial investigation of volatiles by principal component analysis, volatiles were separated on a capillary GC column (TG-SQC, 15 m by 0.25mm; film thickness, Thermo Scientific, UK). The injection volume (splitless) was 1μl, the injector temperature was 200°C, and helium was used as the carrier gas at a constant flow rate of 2.6 ml min^{-1} in an oven maintained at 30°C for 5 minutes and then programmed at 15°$C.min^{-1}$ to 230°C. The column was directly coupled to a mass spectrometer (ISQ LT, Thermo Scientific, UK) with a MS transfer line temperature of 240°C. Ionization was by electron impact with an ion source temperature of 250°C in positive ionization. Mass ions were detected between 30 and 650 m/z. Data were collected using Xcalibur software (Thermo Scientific). Principal component analysis on the mass spectra was performed with MetaboAnalyst 2.0 [60] using binned m/z and per cent total ion count (%TIC) values.

Confirmation of identities of specific organic compounds comprising the blends emitted by mock-inoculated and virus-infected plants was carried out by re-analysis of trapped organic compounds using a Thermo-Finnigan Trace GC directly coupled to a mass spectrometer (MAT-95 XP, Thermo-Finnigan, Bremen, Germany) equipped with a cold on-column injector. Two microliters of collected volatiles were separated on an HP1 capillary gas chromatography column (50 m x 0.32 mm I.D.) in an oven maintained at 30°C for 5 min and then programmed at 5°$C.min^{-1}$ to 250°C [61]. The carrier gas was helium. Ionization was by electron impact at 70 eV at 220°C. Compounds were identified by comparison of spectra with mass spectral databases (National Institute of Standards and Technology: http://www.nist.gov/), as well as by co-injection with authentic standards on a Hewlett-Packard 6890 gas chromatograph with two different columns of different polarity (HP1 and DB-WAX).

Mathematical Modeling

Our model tracks the interaction over evolutionary time between virus resistant and virus susceptible phenotypes in a population of diploid annual plants. The plant population size is assumed to be large and to remain constant over generations. Since CMV is a broad host-range pathogen, we can reasonably make the simplifying assumption that within-generation pathogen prevalence is not affected by the density of resistance in the focal host plant species. The proportion of susceptible plants that become virus infected in each generation is therefore held constant as a parameter (α) in our model. We model resistance as controlled by a single bi-allelic locus, with resistant (R) and susceptible (r) forms, and we assume R is dominant.

We assume infected plants produce fewer seeds, with the parameter δ controlling the proportionate number of viable seeds produced per ovary on a virus-infected plant. We additionally assume that virus resistance carries no fitness penalty when compared to uninfected susceptible hosts. If the reduction in seed number were the only consequence of virus infection, resistance would certainly fix in the plant population under such a conservative assumption on

the cost of virus resistance for the plant. However, we also assume that increased attractiveness to pollinators means infected plants are more likely to reproduce, as both male (pollen donor) and female (seed producing) parents.

In particular, we assume the pollinator density remains constant over generations, and that this pollinator density leads to an average of γ pollinator visits per flower averaged over all plants over the entire reproductive season. We assume that flowers visited by pollinators will certainly be pollinated: by cross-pollination (proportion φ) or by self-pollination (proportion 1 – φ). Self-pollination after a visit by a pollinator can be due to either geitonogamous pollen transfer from flowers on the same plant, or via autogamous buzz-pollination (*cf*. Figs 5 and 6). A proportion σ of the remaining ovules in flowers that are not visited by pollinators also go on to self-pollinate. The potential selective benefit to virus-infected plants is caused by pollinator preference. We assume that an individual pollinator is ν times more likely to visit a flower on an infected vs. an uninfected plant than would be expected by chance alone. This potentially increases female (seed producing) fitness by making ovules on infected plants more likely to be fertilized, and male (pollen donor) fitness by increasing rates of pollen transfer from infected plants.

Given these assumptions, our model tracks the proportion of the plant population in generation n with genotype *RR*, *Rr* or *rr*, which we denote by x_n, y_n and z_n, respectively. The equations linking populations over generations are

$$x_{n+1} = \zeta_n\left(\epsilon_R\left(x_n + \frac{y_n}{4}\right) + \kappa_R\left(\beta_{RR} + \frac{\beta_{Rr}}{2}\right)\left(x_n + \frac{y_n}{2}\right)\right),$$
$$y_{n+1} = \zeta_n\left(\frac{\epsilon_R y_n}{2} + \kappa_R\left(\frac{\beta_{Rr}}{2} + \beta_{rr}\right)x_n + \frac{\kappa_R y_n}{2} + \kappa_r\left(\beta_{RR} + \frac{\beta_{Rr}}{2}\right)z_n\right),$$
$$z_{n+1} = \zeta_n\left(\frac{\epsilon_R y_n}{4} + \epsilon_r z_n + \left(\frac{\beta_{Rr}}{2} + \beta_{rr}\right)\left(\frac{\kappa_R y_n}{2} + \kappa_r z_n\right)\right),$$

in which

$$\eta = \frac{1}{1 + (v-1)\alpha z_n},$$
$$\beta_{RR} = \eta x_n,$$
$$\beta_{Rr} = \eta y_n,$$
$$\beta_{rr} = \eta(1 + (v-1)\alpha)z_n,$$
$$\omega_- = 1 - e^{-\gamma\eta},$$
$$\omega_+ = 1 - e^{-v\gamma\eta},$$
$$\theta_- = (1-\omega_-)\sigma + \omega_-(1-\phi),$$
$$\mu_- = \omega_-\phi,$$
$$\theta_+ = (1-\omega_+)\sigma + \omega_+(1-\phi),$$
$$\mu_+ = \omega_+\phi,$$
$$\epsilon_R = \theta_-,$$
$$\kappa_R = \mu_-,$$
$$\epsilon_r = \alpha\delta\theta_+ + (1-\alpha)\theta_-,$$
$$\kappa_r = \alpha\delta\mu_+ + (1-\alpha)\mu_-$$

and where ζ_n is chosen in each generation to ensure $x_{n+1} + y_{n+1} + z_{n+1} = 1$. A full derivation of the model showing how it follows from the underlying assumptions is given in S1 Text.

The majority of the results presented in the main text are relative to our default parameterization of the model. By default we take the following parameter values: $\nu = 3.0$, $\delta = 0.5$, $\gamma = 1.0$, $\sigma = 0.25$, $\varphi = 0.75$ and = 0.75. However, as described above, we perform a full two-way sensitivity analysis of pairs of parameters around these default values (Fig 8) to test the robustness of our results to our choice of parameterization.

The behaviour of the model can most easily be characterised in terms of which genotypes persist in the long-term. This classification follows from a stability analysis of the susceptible-free (i.e. $x_n = 1$, $y_n = z_n = 0$) and resistance-free (i.e. $x_n = y_n = 0$, $z_n = 1$) equilibria. Since we are working in discrete time, an equilibrium is stable if the magnitude of the largest Eigenvalue of the Jacobian matrix evaluated at the equilibrium is less than unity [62]. If neither equilibrium is stable then both susceptible and resistant plants are able to invade a population consisting almost exclusively of the other when rare, and so the genotypes are predicted to coexist. If only the susceptible-free equilibrium is stable, then resistance dominates. If only the resistance-free equilibrium is stable, then susceptibility dominates. But if both equilibria are stable, then the long term outcome depends on the initial densities of each genotype.

Extensive numerical simulations of the model were performed to verify that local stability analyses could be used to infer the long-term outcome for all initial conditions. In particular we tested 10,000 combinations of parameters and initial conditions (1,000 sets of randomly-chosen parameters, each simulated starting from 10 independent and randomly-selected sets of initial conditions). In all cases the outcome after 10,000 generations of the model matched that predicted by the (purely local) stability analysis described above. We also performed a number of individual tests for pairs of sets of parameters chosen to cross stability boundaries: the stability analysis predicted behaviour in full simulations of the model in the large number of cases we tested.

Supporting Information

S1 Table. Collated free choice bee behavioural assay raw data used for Fig 1B.
(XLSX)

S2 Table. Collated differential conditioning assay raw data used for Fig 2 and Fig 3.
(XLS)

S1 Text. Derivation and additional details of the mathematical modeling.
(PDF)

S1 Fig. Detection and quantification of CMV-Fny and CMVΔ2b RNA in tomato. CMV-Fny accumulates to a higher titer than CMVΔ2b in systemically-infected tomato leaves. (A) Semi-quantitative reverse transcription-polymerase chain reaction (RT-PCR) analysis of viral RNA (*vRNA*) accumulation leaves of tomato plants systemically infected with CMV-Fny (CMV) or CMVΔ2b. Leaf tissue samples were harvested for RNA extraction at 10 and 18 days post-inoculation (dpi). CMV RNA accumulation was determined by RT-PCR after 30 cycles of PCR and compared to the levels of the *elongation factor 1 alpha* (*EF1α*) transcript (to act as an internal loading control). The CMV-specific PCR products from CMV-infected leaves accumulated to higher levels than those from CMVΔ2b infected leaves. (B) RT-quantitative PCR of CMV accumulation relative to CMVΔ2b. Graph shows the mean accumulation of viral RNA in systemically-infected tissues of plants inoculated with CMV-Fny (CMV) or CMVΔ2b (Δ2b) at 10 and 18 dpi. Mean accumulation of virus-specific PCR products is shown for CMV and CMVΔ2b and error bars represent standard errors around the mean for n = 4 samples for CMVΔ2b at 10

dpi and n = 3 and 2, respectively, for CMV at 10 and 18dpi. The housekeeping transcript control was *EF1α* and levels are shown relative to CMVΔ2b, which is designated as '1'.
(PDF)

S2 Fig. Impacts of CMV-Fny on tomato flowering characteristics. (A) The tomato-cucumber mosaic virus (CMV) pathosystem is a virus-plant interaction in which infection with CMV-Fny does not greatly affect the mean number of flowers produced per plant in infected (CMV) versus mock-inoculated (Mock) tomato plants ($n = 6$ plants; one-way ANOVA: $F(1,10) = 0.024$, $p = 0.8803$). (B) Infection with CMV-Fny does not greatly affect the morphology of tomato flowers. Typical flowers from mock-inoculated (Mock) plants and plants infected with CMV-Fny (CMV) are shown. C. Infection with CMV-Fny stunts host growth. The mean height of plants at the point of flowering is shown for plants infected with CMV-Fny (CMV) versus mock-inoculated (Mock) plants ($n = 4$ plants and $n = 3$ plants, respectively; one-way ANOVA: $F(1,5) = 52.92$, $p = 0.0077$) (C). (D) CMV infection accelerated flowering; decreasing time to flowering (days post-sowing; $n = 3$ plants [CMV-Fny] and $n = 4$ plants [Mock]; one-way ANOVA: $F(1,5) = 10.71$, $p = 0.0221$). Mock, mock-inoculated; CMV, infected with CMV-Fny. Asterisks indicate significant differences. Error bars represent the standard error of the mean.
(PDF)

S3 Fig. Characteristics of pollen from flowers of CMV-infected and mock-inoculated tomato plants. (A) Pollen yield from mock-inoculated and virus infected flowers is similar. Fully open flowers from 12 mock-inoculated (mock) and nine CMV-PV0187-infected (CMV) plants were excised into microfuge tubes containing 300μl of water and vortexed for 40 seconds. Using a microscope, released pollen grains were counted in technical triplicates using a cell-counting chamber. The mean number of pollen grains released by flowers is shown. Error bars indicate standard error around the mean. (B) The viability of pollen from mock-inoculated and CMV-infected flowers is similar. Pollen was harvested into microfuge tubes from flowers (at developmental stage 6) by manual buzzing with an electrical toothbrush and stained with fluorescein diacetate. Data are from nine mock-inoculated and nine CMV-PV0187 infected plants. Esterase activity in viable pollen grains releases fluorescein that fluoresces under blue light (Li, X., 2011 http://www.bio-protocol.org/e75) [58] (see panel C). The percentage of pollen grains fluorescing (i.e. viable) is indicated with error bars indicating standard error around the mean. (C) Typical microscopic fields of view for pollen grains extracted from flowers of mock-inoculated (mock) and CMV-PV0187-infected (CMV) plants viewed under blue light and bright field with an epi-fluorescent microscope (DMRXA, Zeiss) connected to a digital camera (DFC425, Zeiss). Upper panels were viewed with blue light illumination under bright field optics enabling viable (fluorescent) and non-viable (non-fluorescent) pollen grains to be counted. Lower panels show pollen grains viewed with epi-fluorescent optics only. Scale bar = 100μm.
(PDF)

S4 Fig. Sequencing and phylogenetic analysis of CMV-PV0187. The three genomic RNAs of CMV-PV0187 were sequenced. The RNA sequences were compared to those of CMV-Fny and other CMV strains and isolates. (A) Phylogenetic analysis using the RNA sequences of CMV-PV0187 RNAs 1, 2, and 3, with corresponding sequences of other CMV strains and isolates. Phylogenetic analysis using the neighbour-joining method under the Kimura-2 parameter was conducted in MEGA software (Version 6.06). The bootstrap consensus tree was carried out with 1000 replications. Panels (left to right) show the phylogenetic analysis of RNAs1, 2 and 3. The CMV-PV0187 sequence data used in this analysis is available at NCBI

under GenBank accession numbers KP165580, KP165581 and KP165582 corresponding to RNA1, RNA2, and RNA3, respectively. PV0187-CMV groups closely with CMV-Fny (indicated with red diamonds), with which it has an overall 99% RNA sequence identity. (B) The predicted 110 residue amino acid sequences of the 2b proteins of CMV-Fny (Fny 2b: upper sequence) and CMV-PV0187 (PV0187 2b: lower sequence) are identical. The amino acid sequences are a virtual translation of the 2b open reading frames of the two CMV strains. The numbers 60, 61, and 110 indicate amino acid residue positions.
(PDF)

S5 Fig. Impact of CMV-PV0187 on tomato plants. The growth and morphology of leaves, flowers and fruit were compared between tomato plants that had been mock-inoculated or infected with CMV-PV0187. Plants or plant organs were photographed and typical images are shown in panels A-E. (A) Tomato plants inoculated with CMV-PV0187 at the seedling stage show marked stunting (right) compared to mock-inoculated plants (left). (B) Mature, expanded leaves of infected (left) and mock-inoculated (right) plants. (C) Young, upper leaves of infected (left) and mock-inoculated (right) plants. (D) Flowers from mock-inoculated (left) and CMV-PV0187 (right) infected plants are similar in appearance and show no gross differences in morphology. (E) Tomato fruits from mock-inoculated plants (left) are larger than those from CMV-PV0187 infected (right) plants. Scale bars = 3 cm.
(PDF)

S6 Fig. Choices and timings for buzz pollination by bumblebees. Bees spent longer buzz-pollinating (sonicating) flowers on CMV-infected tomato plants. Single bees were released into the flight arena containing three mock-inoculated and three CMV-infected flowering tomato plants (Fig 6; S8 Fig). The time each bee spent buzz-pollinating its first five choices of flower was measured using a stopwatch. n = the number of bees making each choice.
(PDF)

S7 Fig. Sensitivity analysis showing discrete time exponential growth rates for a rare mutant type of plant in the vicinity of the equilibrium from which that type is absent. (A) Growth rate of resistant mutants in the vicinity of the (0,0,1) equilibrium at which only susceptible plants are present. The panel shows a series of full two-way sensitivity analyses of the model, showing effects on the growth rate of rare mutant resistant plants in the vicinity of the equilibrium at which only susceptible plants are present, caused by independently changing pairs of parameters (all other parameters fixed). All pair-wise combinations of two parameters are shown: dots on each axis show default values of each parameter. In all cases, the magnitude of the largest Eigenvalue of the Jacobian matrix at the model equilibrium–which is equivalent to the initial discrete time rate of exponential growth over successive seasons of rare mutant resistant plants -is shown by color. Note that Fig 8 in the main text characterises long-term evolutionary outcomes by distinguishing regions in which growth rates of each type of mutant are larger than or smaller than one, and so in which the equilibria can be invaded (or not): these results therefore provide additional numerical detail in support of that figure. (B) Growth rate of susceptible mutant plants in the vicinity of the (1,0,0) equilibrium at which only homozygous resistant plants are present (all other details as per Panel A).
(PDF)

S8 Fig. Arena used for pollination experiments. Design of free choice bee-pollination experiment. (A) A large flight arena (125 x 370 x 90cm: H x W x D) was constructed out of nylon netting with three zipped doors to allow full access. Within this flight arena (zone delineated with a white rectangle) a bumblebee colony was attached by a tube to a small flight arena (38 x 44 x 71 cm; H x W x D) containing a microtiter plate filled with 30% sucrose (not visible) to allow

the bumblebees to feed freely. Sliding gates on the side of the small arena permitted one bee to be released into the larger arena containing three mock-inoculated and three cucumber mosaic virus (CMV)-infected flowering tomato plants. (B) Cartoon demonstrating the arrangement of mock-inoculated (green plants) and CMV-infected plants (red) within the larger flight arena. (PDF)

Acknowledgments

We thank Adrienne Pate, Matthew Dorling and Pete Michna for expert technical assistance, and Alex Webb, Veronica Bennett, Andrea Manica, John Welch, Ruairi Donnelly, Frank van den Bosch, Netsai Mhlanga, Peter Palukaitis and David Baulcombe for useful discussions, and Syngenta-Bioline for the gift of bumblebees for initial experiments. We thank Jim Carrington for *dcl* and *ago* mutant lines.

Author Contributions

Conceived and designed the experiments: JPC BJG HMW JHW SCG AMM NJC.

Performed the experiments: SCG JHW SJ AMM MPD TJAB JCC OJF AR SIR EM.

Analyzed the data: JPC NJC CND BJG SCG AMM MPD TJAB JHW JAP SJ.

Wrote the paper: JPC SCG NJC BJG JHW AMM TJAB JAP.

Carried out mathematical modeling and statistical analyses: NJC CND.

References

1. Klein A-M, Vaissiere BE, Cane JH, Steffan-Dewenter I, Cunningham SA, et al. (2007) Importance of pollinators in changing landscapes for world crops. Proc Royal Soc B 274: 303–313.
2. Knight TM, Steets JA, Vamosi JC, Mazer SJ, Burd M, et al. (2005) Pollen limitation of plant reproduction: pattern and process. Ann Rev Ecol Evol Syst 36: 467–497.
3. Ghazoul J (2006) Floral diversity and the facilitation of pollination. J Ecol 94: 295–304.
4. Hogendoorn K, Gross CL, Sedgley M, Keller MA (2006) Increased tomato yield through pollination by native Australian *Amegilla chlorocyanea* (Hymenoptera: Anthophoridae). J Econ Entomol 99: 828–833. PMID: 16813318
5. Arpaia S, Battafarano R, Chen LY, Devos Y, Di Leo GM, et al. (2012) Assessment of transgene flow in tomato and potential effects of genetically modified tomato expressing Cry3Bb1 toxins on bumblebee feeding behaviour. Ann Appl Biol 161: 151–160.
6. Glover BJ, Bunnewell S, Martin C (2004) Convergent evolution within the genus *Solanum*: the specialised anther cone develops through alternative pathways. Gene 331: 1–7. PMID: 15094186
7. Jacquemond M (2012) Cucumber mosaic virus. Adv Virus Res 84: 439–504. doi: 10.1016/B978-0-12-394314-9.00013-0 PMID: 22682176
8. Palukaitis P, Garcia-Arenal F (2003) Cucumoviruses. Adv Virus Res 62: 241–323. PMID: 14719367
9. Handford MG, Carr JP (2006) Plant metabolism associated with resistance and susceptibility. In: Loebenstein G, Carr JP, editors. Natural Resistance Mechanisms of Plants to Viruses. London: Springer-Verlag. pp. 315–340.
10. Mauck KE, De Moraes CM, Mescher MC (2010) Deceptive chemical signals induced by a plant virus attract insect vectors to inferior hosts. Proc Natl Acad Sci USA 107: 3600–3605. doi: 10.1073/pnas.0907191107 PMID: 20133719
11. Mauck KE, Bosque-Perez NA, Eigenbrode SD, De Moraes CM, Mescher MC (2012) Transmission mechanisms shape pathogen effects on host-vector interactions: evidence from plant viruses. Func Ecol 26: 1162–1175.
12. Raguso RA (2008) Wake up and smell the roses: the ecology and evolution of floral scent. Ann Rev Ecol Evol Syst 39: 549–569.

13. Spaethe J, Tautz J, Chittka L (2001) Visual constraints in foraging bumblebees: flower size and color affect search time and flight behavior. Proc Natl Acad Sci USA 98: 3898–3903. PMID: 11259668
14. Whitney HM, Kolle M, Andrew P, Chittka L, Steiner U, et al. (2009) Floral iridescence, produced by diffractive optics, acts as a cue for animal pollinators. Science 323: 130–133. doi: 10.1126/science.1166256 PMID: 19119235
15. Balkenius A, Rosén W, Kelber A (2006) The relative importance of olfaction and vision in a diurnal and a nocturnal hawkmoth. J Comp Physiol A: Neuroethology Sensory Neural and Behavioral Physiology 192: 431–437.
16. Klahre U, Gurba A, Hermann K, Saxenhofer M, Bossolini E, et al. (2011) Pollinator choice in *Petunia* depends on two major genetic loci for floral scent production. Curr Biol 21: 730–739. doi: 10.1016/j.cub.2011.03.059 PMID: 21497087
17. Kessler D, Diezel C, Baldwin IT (2010) Changing pollinators as a means of escaping herbivores. Curr Biol 20: 237–242. doi: 10.1016/j.cub.2009.11.071 PMID: 20096581
18. Ziebell H, Murphy AM, Groen SC, Tungadi T, Westwood JH, et al. (2011) Cucumber mosaic virus and its 2b RNA silencing suppressor modify plant-aphid interactions in tobacco. Sci Rep 1: 187. doi: 10.1038/srep00187 PMID: 22355702
19. Westwood JH, Groen SC, Du Z, Tungadi T, Lewsey MG, et al. (2013). A trio of viral proteins tunes aphid-plant interactions in *Arabidopsis thaliana*. PlosOne doi: 10.1371/journal.pone.0083066
20. Chen F, Tholl D, D'Auria JC, Farooq A, Pichersky E, et al. (2003) Biosynthesis and emission of terpenoid volatiles from *Arabidopsis* flowers. Plant Cell 15: 481–494. PMID: 12566586
21. Dyer AG, Chittka L (2004) Fine colour discrimination requires differential conditioning in bumblebees. Naturwissenschaften 91: 224–227. PMID: 15146269
22. Whitney HM, Dyer A, Chittka L, Rands SA, Glover BJ (2008) The interaction of temperature and sucrose concentration on foraging preferences in bumblebees. Naturwissenschaften 95: 845–850. doi: 10.1007/s00114-008-0393-9 PMID: 18523748
23. González I, Rakitina D, Semashko M, Taliansky M, Palukaitis P, et al. (2012) RNA binding is more critical to the suppression of silencing function of *Cucumber mosaic virus* 2b protein than nuclear localization. RNA 18: 771–782. doi: 10.1261/rna.031260.111 PMID: 22357910
24. Zhang XR, Yuan YR, Pei Y, Lin SS, Tuschl T, et al. (2006) *Cucumber mosaic virus*-encoded 2b suppressor inhibits *Arabidopsis* ARGONAUTE1 cleavage activity to counter plant defense. Genes Dev 20: 3255–3268. PMID: 17158744
25. Morse A, Kevan P, Shipp L, Khosla S, McGarvey B (2012) The impact of greenhouse tomato (*Solanales*: *Solanaceae*) floral volatiles on bumble bee (*Hymenoptera*: *Apidae*) pollination. Environ Entomol 41:855–864.
26. Harth JE, Winsor JA, Weakland DR, Nowack KJ, Ferrari MJ, et al. (2016) Effects of virus infection on pollen production and pollen performance: Implications for the spread of resistance alleles. Am J Bot 103:577–583. doi: 10.3732/ajb.1500165 PMID: 26905087
27. Fraile A, García-Arenal F (2010) The coevolution of plants and viruses: Resistance and pathogenicity. Adv Virus Res 76:1–32. doi: 10.1016/S0065-3527(10)76001-2 PMID: 20965070
28. Kulahci IG, Dornhaus A, Papaj DR (2008) Multimodal signals enhance decision making in foraging bumble-bees. Proc Royal Soc B 275: 797–802.
29. Kunze J, Gumbert A (2001) The combined effect of color and odor on flower choice behavior of bumblebees in flower mimicry systems. Behav Ecol 12: 447–456.
30. Rodriguez-Saona C, Parra L, Quíroz A, Isaacs R (2011) Variation in highbush blueberry floral volatile profiles as a function of pollination status, cultivar, time of day and flower part: Implications for flower visitation by bees. Ann Bot 107: 1377–1390. doi: 10.1093/aob/mcr077 PMID: 21498566
31. Hull R (2002) Matthews' Plant Virology, 4th Edition. Amsterdam, the Netherlands: Elsevier.
32. Eberle CA, Forcella F, Gesch R, Weyers S, Peterson D, et al. (2014) Flowering dynamics and pollinator visitation of oilseed Echium (*Echium plantagineum*). PLoS ONE 9(11): e113556. doi: 10.1371/journal.pone.0113556 PMID: 25427071
33. Mitchell RJ, Flanagan RJ, Brown BJ, Waser NM, Karron JD (2009) New frontiers in competition for pollination. Ann Bot 103: 1403–1413. doi: 10.1093/aob/mcp062 PMID: 19304814
34. Roossinck MJ (2011) The good viruses: viral mutualistic symbioses. Nature Rev Microbiol 9: 99–108.
35. Xu P, Chen F, Mannas JP, Feldman T, Sumner LW, et al. (2008) Virus infection improves drought tolerance. New Phytol 180: 911–921. doi: 10.1111/j.1469-8137.2008.02627.x PMID: 18823313
36. Westwood JH, McCann L, Naish M, Dixon H, Murphy AM, et al. (2013) A viral RNA silencing suppressor interferes with abscisic acid-mediated signalling and induces drought tolerance in *Arabidopsis thaliana*. Molec Plant Pathol 14: 158–170.

37. Pan H, Chen G, Li F, Wu Q, Wang S, et al. (2013) Tomato spotted wilt virus infection reduces the fitness of a nonvector herbivore on pepper. J Econ Entomol 106: 924–928. PMID: 23786083
38. van Molken T, de Caluwe H, Hordijk CA, Leon-Reyes A, Snoeren TAL, et al. (2012) Virus infection decreases attractiveness of white clover plants for a non-vectoring herbivore. Oecologia 170: 433–444. doi: 10.1007/s00442-012-2322-z PMID: 22526939
39. Mauck KE, De Moraes CM, Mescher MC (2010) Effects of *Cucumber mosaic virus* infection on vector and non-vector herbivores of squash. Comm Integ Biol 3:579–582.
40. Gibbs AJ (1980) A plant virus that partially protects its wild legume host against herbivores. Intervirology 13:42–47. PMID: 7358509
41. Dawkins R (1982) The Extended Phenotype: the Long Reach of the Gene. Oxford: Oxford University Press.
42. Karasov TL, Kniskern JM, Gao L, DeYoung BJ, Ding J et al. (2014) The long-term maintenance of a resistance polymorphism through diffuse interactions. Nature 512:436–440. doi: 10.1038/nature13439 PMID: 25043057
43. Caicedo AL, Schaal BA (2004) Heterogeneous evolutionary processes affect R gene diversity in natural populations of *Solanum pimpinellifolium*. Proc Natl Acad Sci USA 101:17444–17449. PMID: 15583124
44. Benjamin A, McCallum B (2008) A World Without Bees. Bradford: Guardian Books. 256 p.
45. Potts SG, Biesmeijer JC, Kremen C, Neumann P, Schweiger O, et al. (2010) Global pollinator declines: trends, impacts and drivers. Trends Ecol Evol 25: 345–353. doi: 10.1016/j.tree.2010.01.007 PMID: 20188434
46. Gill RJ, Ramos-Rodriguez O, Raine NE (2012) Combined pesticide exposure severely affects individual- and colony-level traits in bees. Nature 491: 105–108. doi: 10.1038/nature11585 PMID: 23086150
47. Roossinck MJ, Palukaitis P (1990) Rapid induction and severity of symptoms in zucchini squash (*Cucurbita pepo*) map to RNA1 of cucumber mosaic virus. Molec Plant-Microbe Interact 3: 188–192.
48. Ryabov EV, Fraser G, Mayo MA, Barker H, Taliansky M (2001) Umbravirus gene expression helps *Potato leafroll virus* to invade mesophyll tissues and to be transmitted mechanically between plants. Virology 286: 363–372. PMID: 11485404
49. Lewsey M, Robertson FC, Canto T, Palukaitis P, Carr JP (2007) Selective targeting of miRNA-regulated plant development by a viral counter-silencing protein. Plant J 50: 240–252. PMID: 17444907
50. Morel JB, Godon C, Mourrain P, Beclin C, Boutet S, et al. (2002) Fertile hypomorphic *ARGONAUTE* (*ago1*) mutants impaired in post-transcriptional gene silencing and virus resistance. Plant Cell 14: 629–639. PMID: 11910010
51. Finnegan EJ, Margis R, Waterhouse PM (2003) Posttranscriptional gene silencing is not compromised in the *Arabidopsis CARPEL FACTORY* (*DICER-LIKE1*) mutant, a homolog of Dicer-1 from *Drosophila*. Curr Biol 13: 236–240. PMID: 12573220
52. Harvey JJW, Lewsey MG, Patel K, Heimstädt S, Westwood JH, et al. (2011) An antiviral defence role of AGO2 in plants. PLoS One 6(1): e14639. doi: 10.1371/journal.pone.0014639 PMID: 21305057
53. Livak KJ, Schmittgen TD (2001) Analysis of relative gene expression data using real-time quantitative PCR and the $2^{-\Delta\Delta C}{}_T$ method. Methods 25: 402–408. PMID: 11846609
54. Chittka L, Raine NE (2006) Recognition of flowers by pollinators. Curr Opin Plant Biol 9: 428–435. PMID: 16713328
55. Foster JJ, Sharkey CR, Gaworska AWA, Roberts NW, Whitney HM, Partridge JC (2014) Bumblebees learn polarization patterns. Curr Biol 24:1415–1420. doi: 10.1016/j.cub.2014.05.007 PMID: 24909321
56. Hosmer DW, Lemeshow SK (1989) Applied Logistic Regression. New York: Wiley.
57. Dalgaard P 2008. Introductory statistics with R. Second Edition. New York: Springer.
58. Li X (2011) Pollen Fertility/viability Assay Using FDA Staining. *Bio-protocol* Bio 101: e75. http://www.bio-protocol.org/e75
59. Beale MH, Birkett MA, Bruce TJA, Chamberlain K, Field LM, et al. (2006) Aphid alarm pheromone produced by transgenic plants affects aphid and parasitoid behavior. Proc Natl Acad Sci USA 103:10509–10513. PMID: 16798877
60. Xia J, Mandal R, Sinelnikov I, Broadhurst D, Wishart DS (2012) MetaboAnalyst 2.0—a comprehensive server for metabolomic data analysis. Nucl Acids Res 40: W127–W133 doi: 10.1093/nar/gks374 PMID: 22553367
61. Bruce TJA, Matthes MC, Chamberlain K, Woodcock CM, Mohib A, et al. (2008) *cis*-Jasmone induces *Arabidopsis* genes that affect the chemical ecology of multitrophic interactions with aphids and their parasitoids. Proc Natl Acad Sci USA 105: 4553–4558. doi: 10.1073/pnas.0710305105 PMID: 18356298
62. Britton NF (2003) Essential mathematical biology. London: Springer-Verlag.
